# New Derivatives of 5-((1-Methyl-Pyrrol-2-yl) Methyl)-4-(Naphthalen-1-yl)-1,2,4-Triazoline-3-Thione and Its Coordination Compounds with Anticancer Activity

**DOI:** 10.3390/ijms23169162

**Published:** 2022-08-15

**Authors:** Agnieszka Czylkowska, Suneel Lanka, Małgorzata Szczesio, Kamila Czarnecka, Paweł Szymański, Monika Pitucha, Aneta Drabińska, Bruno Cury Camargo, Jacek Szczytko

**Affiliations:** 1 Institute of General and Ecological Chemistry, Faculty of Chemistry, Lodz University of Technology, Zeromskiego 116, 90-924 Lodz, Poland; 2 Department of Pharmaceutical Chemistry, Drug Analyses and Radiopharmacy, Faculty of Pharmacy, Medical University of Lodz, Muszynskiego 1, 90-151 Lodz, Poland; 3 Department of Radiobiology and Radiation Protection, Military Institute of Hygiene and Epidemiology, 4 Kozielska St., 01-163 Warsaw, Poland; 4 Independent Radiopharmacy Unit, Faculty of Pharmacy, Medical University of Lublin, Chodzki 4A, 20-093 Lublin, Poland; 5 Faculty of Physics, University of Warsaw, Pasteura 5, 02-093 Warszawa, Poland; 6 Institute of Experimental Physics, Faculty of Physics, University of Warsaw, Pasteura 5, 02-093 Warszawa, Poland

**Keywords:** coordination compounds, anticancer activities, ADMET, molecular docking

## Abstract

A new ligand 5-((1-methyl-pyrrol-2-yl) methyl)-4-(naphthalen-1-yl)-1,2,4-triazoline-3-thione (C15) and its metal complexes with formulae: Mn(C15)Cl_2_MeOH (**1**), Fe(C15)Cl_2_MeOH (**2**), Ni(C15)Cl_2_MeOH (**3**), Cu(C15)_2_Cl_2_ (**4**) and Zn(C15)_4_Cl_2_ (**5**) have been synthesized. The C15 ligand and complexes were characterized by NMR, elemental analysis, FT-IR, EPR, magnetic and TGA studies. The anticancer activities of the organic ligand (C15) and complexes (**1**–**5**) were evaluated against human colon adenocarcinoma (HT29) and human lung (A549) cancer cell lines. The complex (**1**) exhibited potential activity at concentration of 794.37 μM (A549) and 654.31 μM (HT29) in both cancer cells. The complex (**3**) showed significant activity against the HT29 cancer cell line with an IC_50_ value of 1064.05 μM. This article highlights some of the metals that have become important in the development of new coordination complexes and the treatment of cancer. Additionally, for C15, the toxicity was predicted by ADMET analysis and molecular docking.

## 1. Introduction

Cancer is the second leading cause of death worldwide. It is predicted that by 2030, the number of new cancer cases will increase to approximately 26 million. Lung cancer is most common in men, second only to breast cancer in women, and is known to have the lowest survival rate [1]. Moreover, colorectal cancer ranks the second and the third in terms of cases diagnosed in women and men, respectively [2]. Due to the designed innovative structure of newly synthesized chemical compounds, they have a chance to act against cancer. For this reason, we decided to use two of the six most commonly diagnosed and leading causes of death from cancer: lung cancer and colon cancer [3].

The current range of active anticancer drugs is very broad, targeting a wide range of cellular and biological properties in many tumour types. Over the last five decades, the development of anticancer drugs has shifted from traditional cytotoxicity to selective agents rationally designed to act on specific cellular targets [4,5]. However, significant challenges remain, and the interface between structural biology and chemistry may be the most fruitful avenue for discovering and improving new anticancer drugs [6]. In nature, many biological systems make extensive use of metal ions, such as zinc (Zn) and copper (Cu), which play an important role in the smooth functioning of living organisms. Transition metals such as copper (Cu), iron (Fe) and manganese (Mn) are involved in a variety of biological processes, from electron transfer through catalysis to patterning, and are often associated with active sites in proteins and enzymes [7]. Dysregulation of some of these essential metals has been implicated in the development of various pathological diseases, such as cancer, during normal biochemical processing [8]. These cellular functions require only trace elements, but in controlled amounts. In contrast, other metals such as arsenic (As), cadmium (Cd), chromium (Cr) and nickel (Ni) are less favourable due to their significant toxic effects, including carcinogenicity [7,9]. The metal ions also influence the biological properties of coordination compounds [10,11,12].

**Figure 1:**
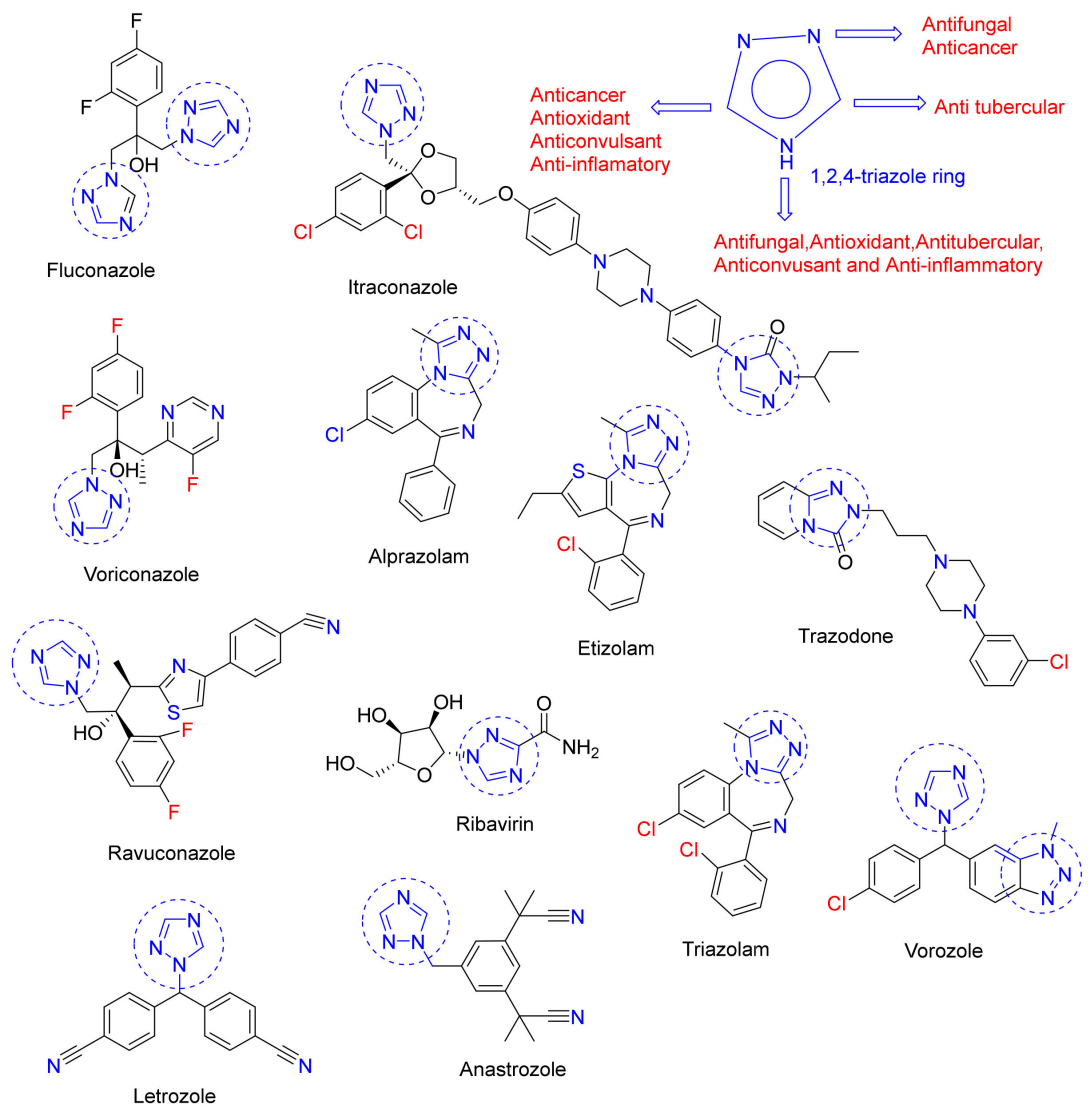
Drugs containing a 1,2,4-triazole ring are available in their therapeutic library.

Heterocyclic compounds, whether natural or synthetic, are endowed with biological properties [13,14,15]. The properties of five-membered heterocyclic systems with three heteroatoms at symmetric positions were extensively studied [16,17,18,19,20,21]. The chemistry of 1,2,4-triazoles is fascinating for heterocycles and their derivatives, especially those belonging to the isothiocyanate class [22,23,24,25,26,27,28]. Heterocyclic compounds are known to play a significant role in the design of a new class of structural entities for medicinal and biological purposes [29,30]. Important pharmacologicals of heterocyclic compounds include 1,2,4-triazoles and their derivatives, which have received much attention because of their interesting biological activities [31,32,33,34,35,36,37,38,39,40,41,42,43]. 1,2,4-Triazoles are present as a unit in a variety of drugs marketed as antineoplastic [44], antifungal [45], antiviral [46], sedative stimulants [46,47,48], anti-inflammatory [49] and antimycotics agents [50] Triazole-based drugs are shown in Figure 1. The conception and tuning of ligand capabilities can ultimately lead to an innovative area of metal chemistry [51,52,53].

On the contrary, coordination chemistry appears to be an important topic because it involves the synthesis of different ligands containing different functional groups and coordinated to metal ions of different oxidation states, which present different geometries with unique properties, novel reactivity, and synergistic effects. It is commonly known that antitumor drugs based on transition metal complexes are less toxic compared, for example, with free ligands. Coordination compounds with d^n^-electron metals are promising antitumor therapeutic agents. Metal ions that take advantage of their unique physiochemical properties have been utilized as powerful tools in cancer diagnosis [54].

In this paper, we focus on 5-((1-methyl-pyrrol-2-yl) methyl)-4-(naphthalen-1-yl)-1,2,4-triazoline-3-thione and its coordination compounds. We choose the naphthyl substituent because of its critical anticancer activity [55,56]. In continuation of the development of this subject, we focus here on filling the research gap on the coordination chemistry of disubstituted 1,2,4-triazole-3-thione derivatives and their chemical, physical and biological properties. In this work, we describe the synthesis and characterization of a new series of metal complexes containing 5-((1-methyl-pyrrol-2-yl) methyl)-4-(naphthalen-1-yl)-1,2,4-triazoline-3-thione. The characterization of these compounds and the results obtained are discussed in this paper.

## 2. Results and Discussion

### 2.1. Synthesis

**Scheme 1:**
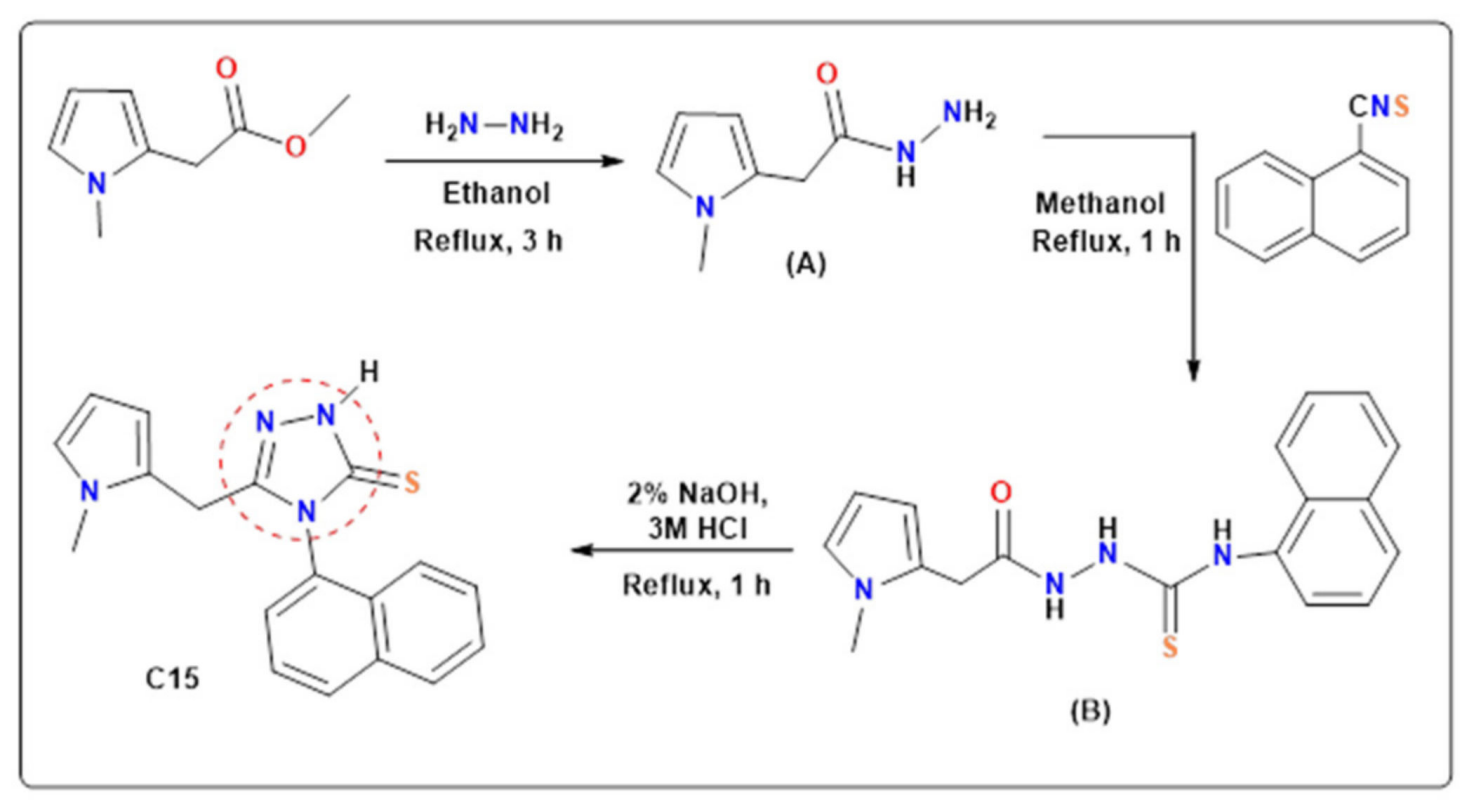
Synthesis of 5-((1-methyl-pyrrol-2-yl)methyl)-4-(naphthalen-1-yl)-1,2,4-triazoline-3-thione.

The disubstituted 1,2,4-triazole-3-thione heterocyclic ligand was synthesized via a multistep reaction using suitable substrates. The substrate for ligand synthesis was 1-methylpyrrole-2-acetate, which was converted to the corresponding hydrazide (A) by reaction with hydrazine hydrate. The hydrazide was reacted with 1-naphthyl isothiocyanate. The reaction was carried out in methanol by heating the materials for 1 h. The 1-(1-methylpyrrol-2-yl) acetyl-4-(1-naphtyl) thiosemicarbazide (B) was subjected to cyclization in a 2% NaOH environment by heating the sample for 2 h. The final compound (C15) resulted from the precipitation of 3M HCl. The schematic representation of synthesis is shown in Scheme 1.

### 2.2. FT-IR Spectra

During complexation, the vibrational mode of the free ligand is changed. Comparing the spectra of 1,2,4-triazole and complexes, it can be seen that the fundamental ν(NH) vibration stretching modes derivate from the triazole ring, which occurs in free ligands in the range of 3094–2762 cm^−1^, and is not changed in coordination compounds. It shows that the NH group in all complexes does not participate in binding with metal ions. In the spectra of the uncoordinated donor, vibrations modes of ν(C = N) and ν(C = C) are visible in the ranges of 1701–1563 and 1509–1403 cm^−1^, respectively. As a result of coordination between the metal ions and the ligand, these frequencies shift towards higher or lower frequencies with peaks for (**1**): 1630, 1610, 1563, 1489, 1403 cm^−1^, for (**2**): 1636, 1613, 1572, 1495, 1412 cm^−1^, for (**3**): 1629, 1603, 1572, 1494, 1413 cm^−1^, for (**4**): 1701, 1631, 1596, 1509, 1468, 1436, 1406 cm^−1^ and for (**5**): 1632, 1572, 1495, 1412 cm^−1^. For all complexes, we can also observe bands in the ranges of 1312–1088 and 806–661 cm^−1^ that correspond to β(CH) and γ(CH) modes, respectively. In the spectra of free ligands, vibration modes of ν(C=S) appear at 1007 and 958 cm^−1^. These bands in the FT–IR spectra of complexes change. The ligand adopts thione form in the complex, which is supported by the presence of *ν*(NH) vibration stretching modes [57,58]. These changes indicate bonds between metal and organic ligands. Additionally, in the spectra of (**1**), (**2**) and (**3**) there are peaks between 3522 and 3190 cm^−1^ characteristics for methanol molecules. By analysing the presented spectra, it can be concluded that in the case of all complexes, the coordination takes place via the sulphur atom contained in the 1,2,4-triazole ring. 

### 2.3. Magnetic Study

All samples presented a paramagnetic-like behaviour above 75 K, denoted by almost-constant χT vs. T curves. However, only (**4**) was well-described as being composed by non-interacting paramagnetic centres with magnetic moment J≠0. We elaborate below.

**Figure 2:**
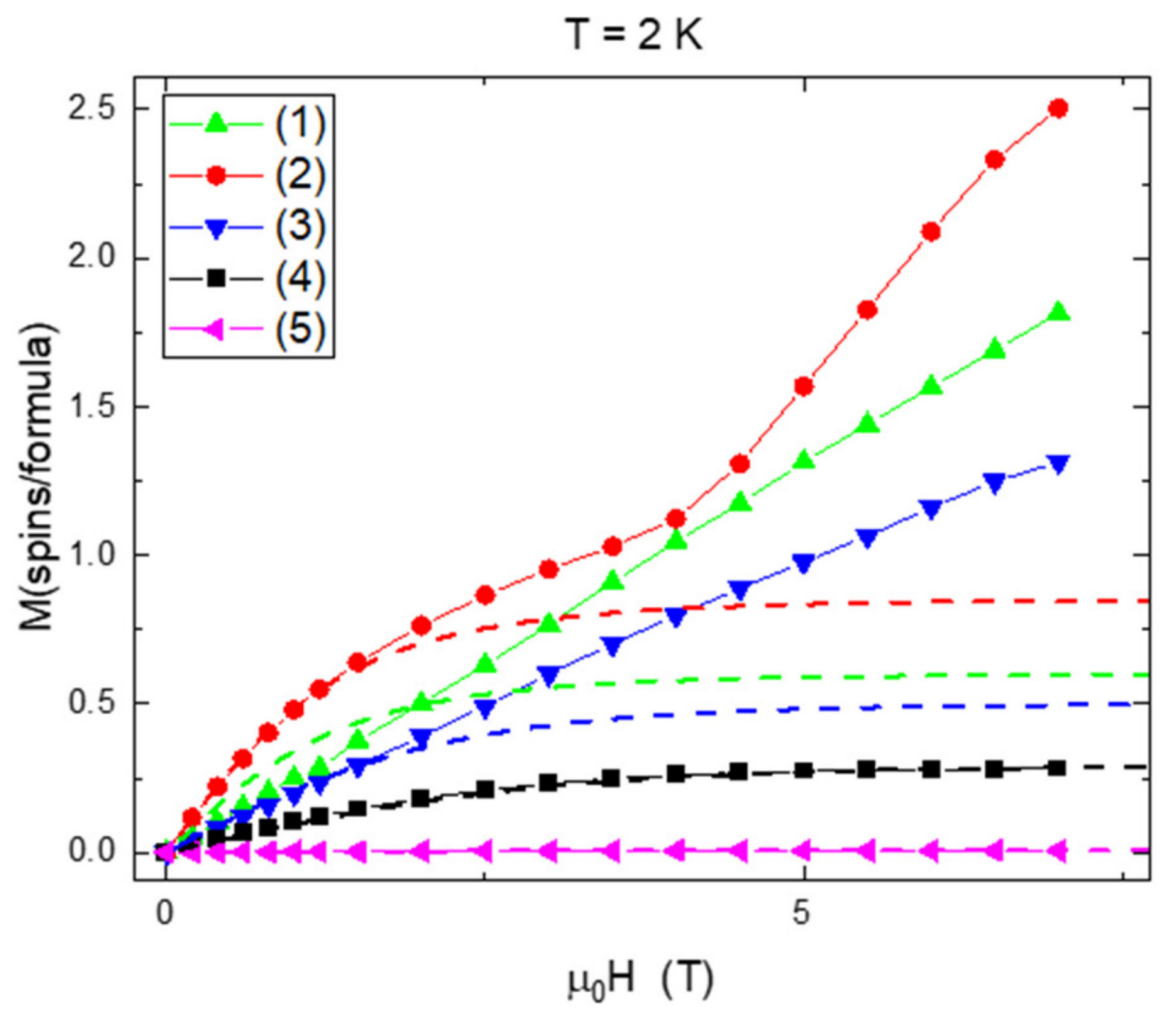
M(H) measurement for complexes (**1**), (**2**), (**3**), (**4**) and (**5**), in units of Bohr magnetons per molecule, obtained at *T* = 2 K. The dashed lines are results expected for non-interacting magnetic moments $y\propto B_{J}\left(x\right)$, *B_J_* the Brillouin function with $x=gJ\mu_{B}k_{B}^{-1}B/T$ and *J* = 1, 0.5, 1, 2 and 2.5 for Zn, Cu, Ni, Fe and Mn, respectively. g is taken at g = 2.0 for all samples. Note that only the X = (**4**) sample is well-described by the dashed line. Samples (**1**), (**2**), (**3**) did not show a saturation of their magnetization, up to $\mu_{0}H\sim 7$ T.

Results for the compounds (**4**) and (**5**) showed magnetic responses corresponding to less than one Bohr magneton per molecule. Data for (**5**) were consistent with a non-magnetic material containing a single magnetic ion per 1000 molecules. Such a small value can be attributed to foreign elements and indicates a lack of magnetism, as is expected for non-interacting Zn(II) magnetic centres, which possess completely-filled d-shell orbitals. Results for the compound (**4**) at *T* < 10 K, on the other hand, revealed a M(H) response well-described by the Brillouin function *B_j_*(*x*) (Figure 2). Such a function suggests that this material contains non-interacting magnetic ions [59], the behaviour of which was consistent with molecules of magnetic moment *J* = 0.5 constituting 30% of the specimen. The extracted value of *J* indicates a Cu(II) oxidation state, corroborating the EPR measurements shown in Section 2.4.

**Figure 3:**
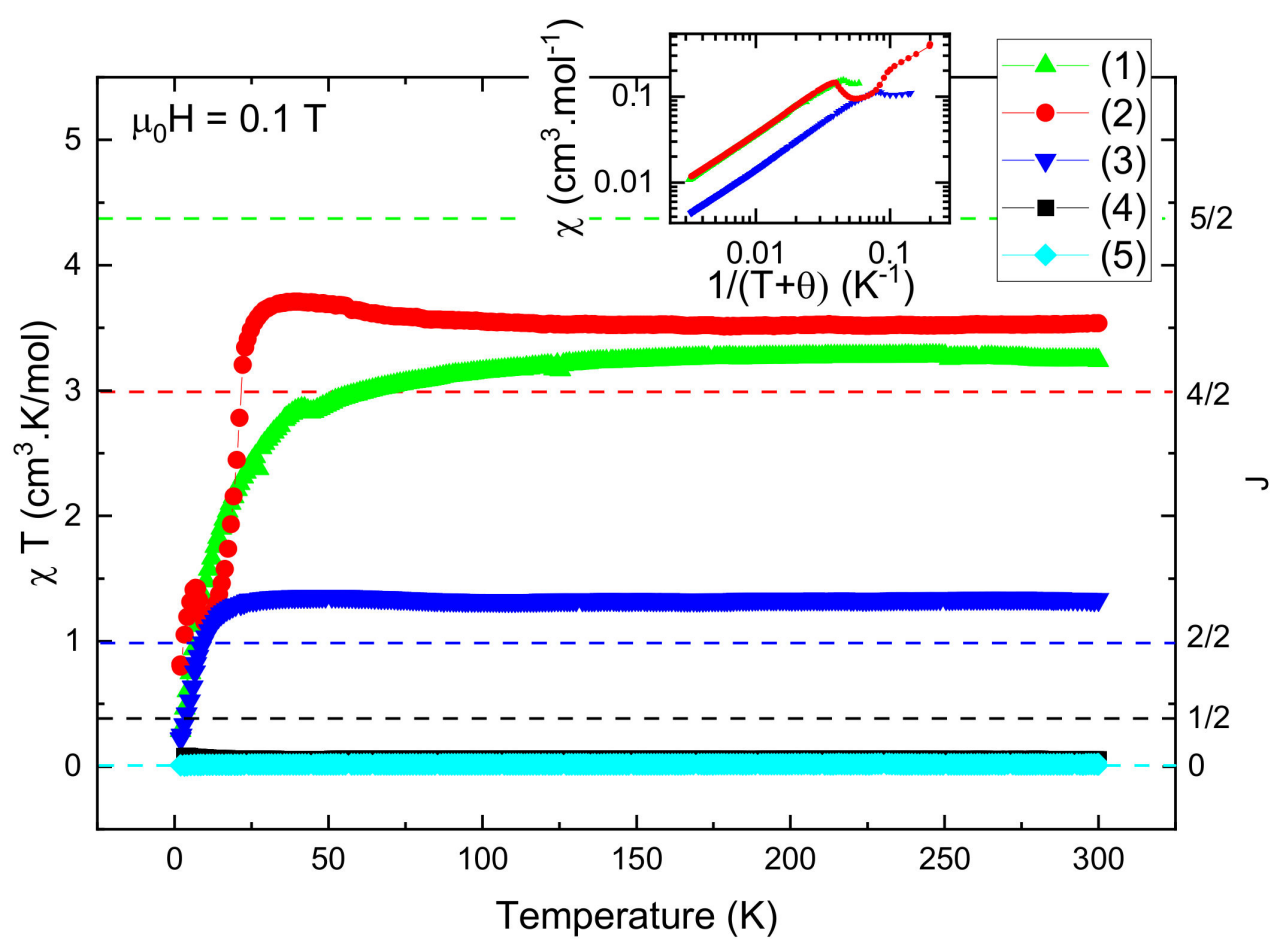
Reduced magnetic response χT vs. T for all samples, measured at $\mu_{0}H=0.1$ T. The horizontal dashed lines represent the expected behaviour for the compound of corresponding colour, had their magnetic response been solely generated by its isolated paramagnetic ion in the (II) oxidation state. The right-hand axis indicates the associated values of *J*. The deviation of the data towards zero, observed below 75 K, indicates antiferromagnetic interactions. The inset shows a plot of χ vs. (*T* + θ)^−1^ for (**1**), (**2**) and (**3**). Here θ is the Curie-Weiss temperature, and equals to 15 K, 3 K and 5 K for (**1**), (**2**) and (**3**), respectively. In this scale, linearity denotes a Curie-Weiss behavior, whereas a deviation towards lower values at large (*T* + θ)^−1^ indicates the presence of a magnetic transition towards an antiferromagnetic state at low temperatures.

Complexes (**1**), (**2**) and (**3**), on the other hand, yielded a non-negligible magnetization, albeit not presenting typical Curie-like behaviour. Instead, the magnetic susceptibility of these compounds could be well-described as χT~ cte for *T* > 100 K (Figure 3) with a deviation towards zero for *T* < 75 K and a sharp transition to a lower magnetization state at lower temperatures—thus indicating predominantly antiferromagnetic interactions. This observation is supported by magnetization vs. magnetic field (M vs. H) measurements up to 7 T, which did not exhibit saturation, even at the lowest temperatures (*T* = 2 K). Such results are presented in Figure 3, which shows measured data together with curves expected for non-interacting magnetic ions constituting a fraction of the sample. A fit of $\chi \left(T\right)$ through a function of the type $\chi \sim 1/\left(T+\theta \right)$ yielded a Weiss temperature θ of 15 K for compound (**1**) (Mn), 3 K for (**2**) (Fe) and 5 K for (**3**) (Ni). These results further demonstrate that magnetic intermolecular interactions are non-negligible, and these materials in a crystalline form cannot be treated as uniformly discrete complexes. Deviations of $\chi T\;\left(T=300\;K\right)$ from those of the corresponding isolated metallic ions vary depending on the compound considered (Figure 3). This suggests that intermolecular magnetic coupling occurs between d-shell electrons of the metallic elements, supporting their role in the compound’s apparent values of *J*. The enhancement of *J* with respect to the isolated magnetic centres is expected in the context of dimer chains and non-isotropic crystalline fields in solids [59] However, a quantitative estimation cannot be obtained in the absence of complex molecular simulations and an accurate determination of the crystallographic environment surrounding the magnetic ions, which escapes the scope of the current work. 

### 2.4. EPR Spectra

**Figure 4:**
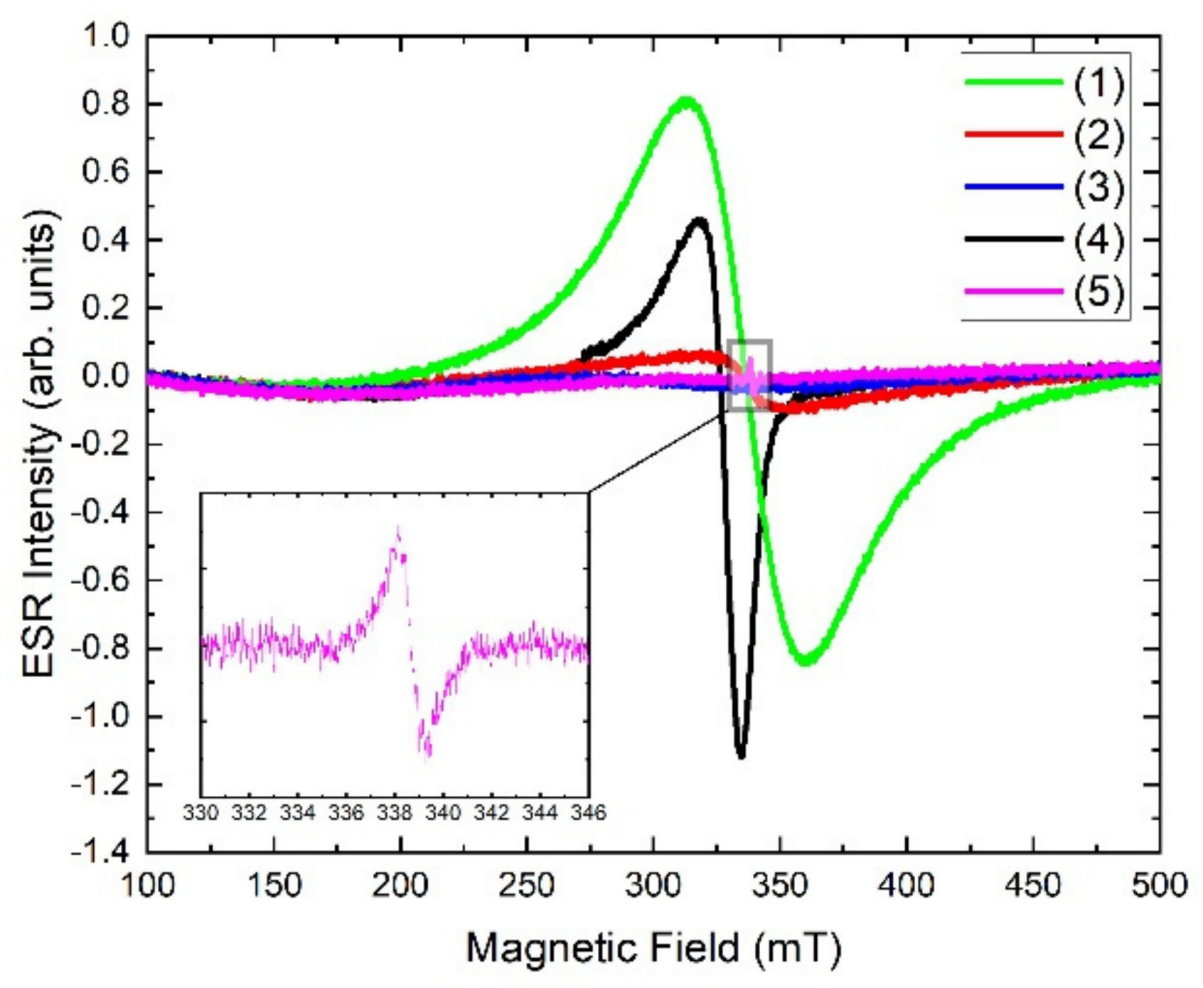
EPR spectra of all complexes (**1**–**5**) at room temperature recalculated to 9.5 GHz. Inset shows the magnification of the sharp signal for complex (**5**).

The EPR spectra of all complexes (**1**–**5**) are presented in Figure 4. The most prominent signal is observed for Mn(II) ions. Surprisingly, the signal consists of a single broad line without any trace of fine or hyperfine structure. The line has a Gaussian shape, so it indicates the isotropic g-factor. This has been observed before in powdered samples at room temperature and was attributed to the existence of dipolar interactions between manganese ions and distortion of their symmetry [60]. Similar broad lines, but with significantly smaller amplitude, were observed also for Fe(II) and Ni(II) ions. The obtained g-factors are listed in Table 1. The substantial difference is observed for complexes (**4**) and (**5**). In the case of Zn(II) ions, the observed line has a Lorentzian shape and P-P width over one order of magnitude smaller. This would indicate the presence of EPR active ions in the studied powder with significantly smaller interactions between them. For compound (**4**), the experimental spectrum shows anisotropy of g-factor. The trend $g_{∥}>g_{⊥}>g_{e}$ suggests $d_{x^{2}-y^{2}}$ ground state for the Cu(II) ion and a tetragonal distortion around the copper(II) ions [61,62,63]. The calculated $G=\frac{g_{∥}-2.0023}{g_{⊥}-2.0023}\approx 4.98$ suggests a negligible exchange interaction between Cu(II) centres [61,64].

### 2.5. Thermal Decomposition of Complexes

**Figure 5:**
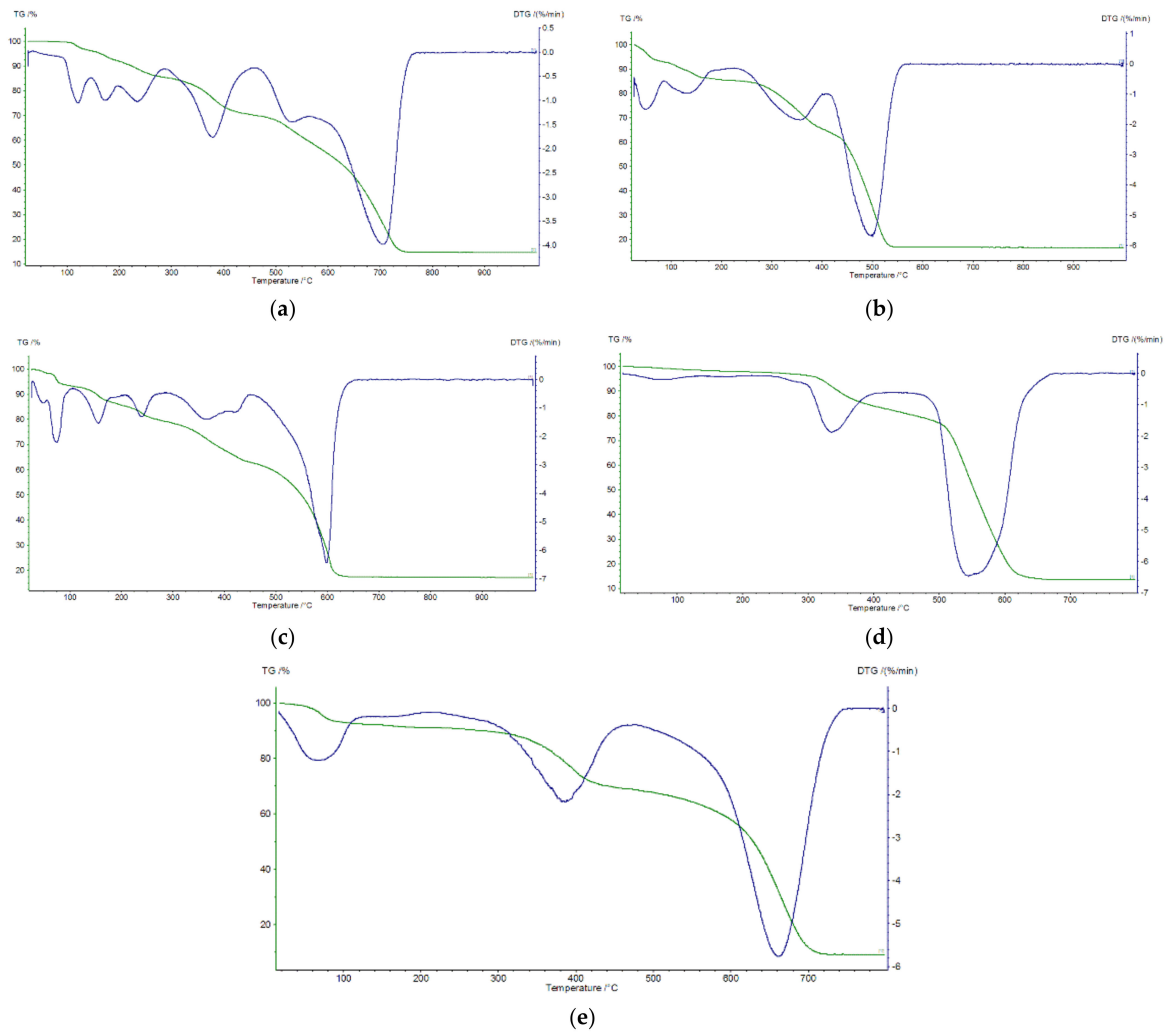
(**a**) TG/DTG curve of compound (**1**); (**b**) TG/DTG curve of compound (**2**); (**c**) TG/DTG curve of compound (**3**); (**d**) TG/DTG curve of compound (**4**); (**e**) TG/DTG curve of compound (**5**).

The thermal decomposition of complexes in the air is a multi-step process. The solid intermediates of the thermolysis products were calculated from the TG and DTG curves. The curves for complexes (**1**), (**2**), (**3**), (**4**) and (**5**) are shown in Figure 5a–e, respectively. All complexes were stable at room temperature and decompose progressively. 

From the TG and DTG curves of complex (**1**), in the temperature range 125–525 °C, the first mass loss is observed. One molecule of methanol and one 1-naphthyl fragment are lost (found. 33.0%, calcd. 33.28%). Next, further decomposition begins. Above 750 °C Mn_2_O_3_ appears (found.: 16.5%, calcd. 16.50%) (Figure 5a). Complex (**2**) is stable at room temperature. It starts to decompose at 50 °C, losing solvent and naphthyl fragments. The loss of mass is 33.0% and calculated as 33.21%. When the temperature rises further, pyrolysis takes place., Thermal decomposition finishes at 550 °C, and as final decomposition product Fe_2_O_3_ is formed (found.: 17.0, calcd.: 16.66) (Figure 5b). For compound (**3**), thermal decomposition starts at 50 °C. The mass loss (found. 34.0%, calcd. 33.02%) occurs at 50–450 °C, which corresponds to the loss of one molecule of methanol and naphthyl. Next, further decomposition of organic molecules takes place. A plateau for NiO on the TG curve is above 650 °C (found: 16.0, calcd.: 15.49) (Figure 5c). Complex (**4**) is the most stable complex; it begins to decompose at 300 °C. The TG curve shows only two steps. The first relates to losing one mol of naphthyl (found. 17.0% calcd.: 16.80%), and the second to the appearance of CuO above 670 °C (found: 18.7%, calcd.: 20.50%) **(**Figure 5d). For compound (**5**), the decomposition process starts at 60 °C. When the temperature increases, partial destruction of organic ligands starts. Then, complete decomposition and combustion of the organic residues takes place. A constant mass level for ZnO begins at 760 °C (found.: 8.0, calcd.: 6.04) (Figure 5e).

### 2.6. PXRD Analysis for Complexes (1), (2) and (3)

**Figure 6:**
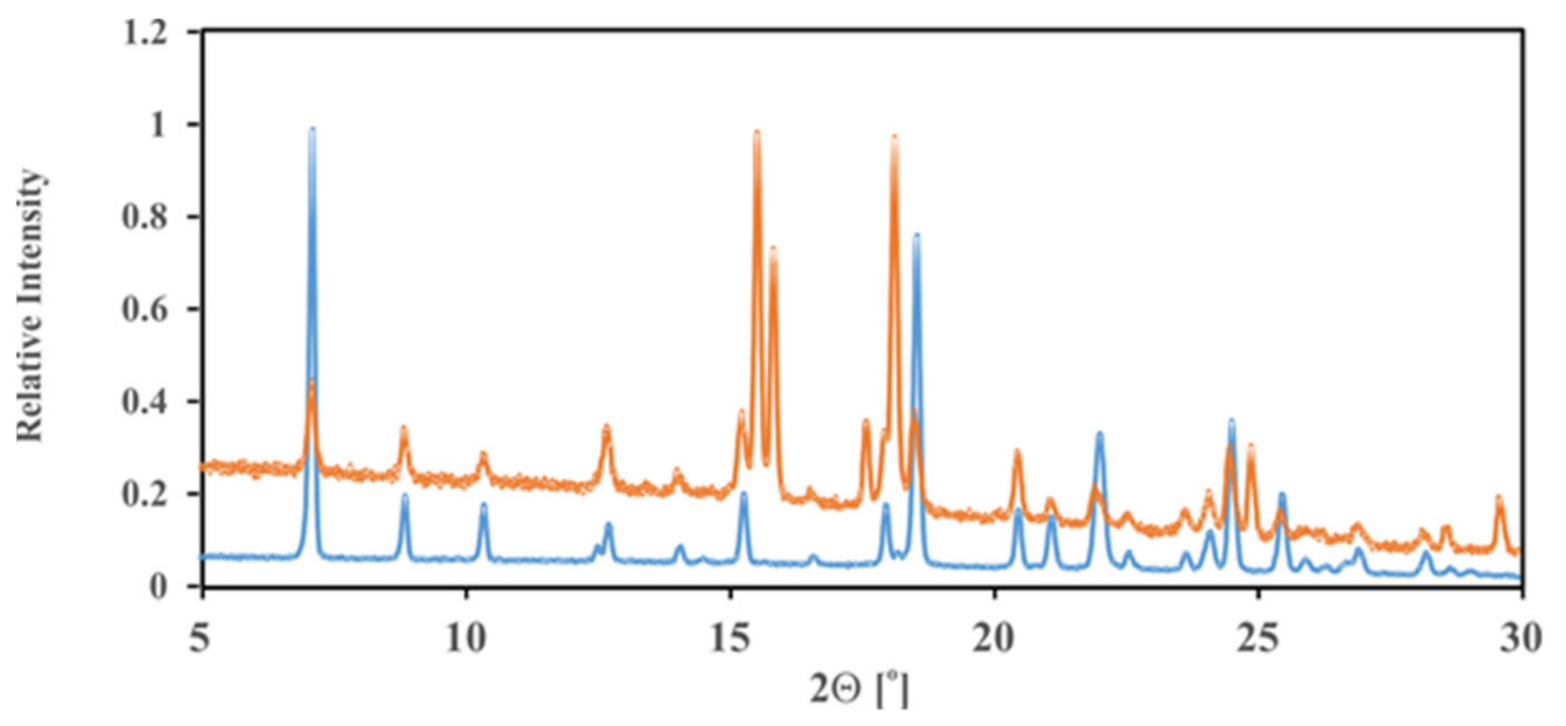
X-ray powder pattern recorded in Bragg Brentano geometry with a Cu Kα (λ = 1.5425 Å) source for compounds (**1**) (blue) and (**3**) (orange) and at room temperature.

The obtained materials did not allow us to perform single crystal X-ray measurements, therefore, the application of the PX-RD technique was our choice. We performed PX-RD analysis at ambient temperature for all studied objects. The (**2**) complex did not show any crystalline reflexes and only an amorphous background was detected. Fortunately, the (**1**) and (**3**) complexes allowed us to obtain a good-quality PX-RD pattern (Figure 6). Our measurements clearly proved that most of the low angle reflexes overlay between (**1**) and (**3**) samples. This means that the shape and unit cell parameters are the same for both complexes. This is probably because they are isostructural or very similar structures. Despite numerous attempts at finding an indexing parameter, it was not possible to determine the crystallographic parameters.

### 2.7. Cell Line Cytotoxicity Predictor: In Silico Prediction of Cytotoxicity for Tumour

Cell line cytotoxicity predictor (CLC-Pred) is a service for the prediction of chemical compounds’ cytotoxic effects. C15 was tested in silico for antitumor activity (Table 2). The analysis indicated the probability of activity on pancreatic, urinary tract, colon and lung cancer. The probability of activity in colon carcinoma is over 35%, while in lung cancer, over 25%, which may indicate the success of the accepted thesis about the activity of the tested compound on these human organs.

### 2.8. ADMET Analysis

**Figure 7:**
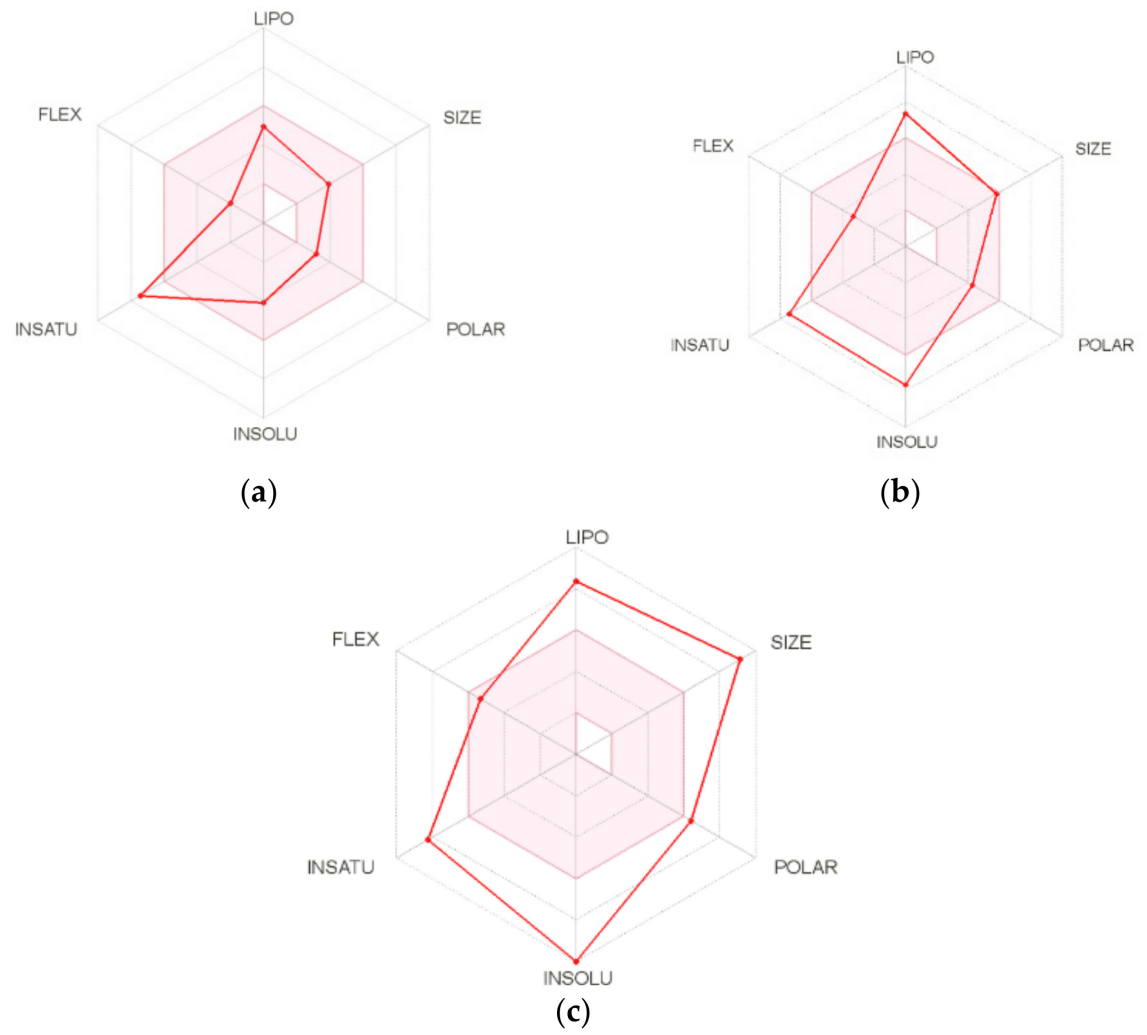
Bioavailability radar for (**a**). C15, (**b**). (**1**) and (**c**). (**4**). (lipophility (LIPO) are within the range −0.7 < XlogP3 < +5.0; molecular weight (SIZE) is 150 g/mol < MW < 500 g/mol; polarity (POLAR) is 20 Å ^2^ < TPSA < 130 Å ^2^; insolubility (INSOLU) is 0 < logS < 6; – insaturation (INSATU) are 0.25 < Fraction Csp3 < 1; and Flexibility (FLEX) are 0 < Num. rotatable bonds < 9).

**Figure 8:**
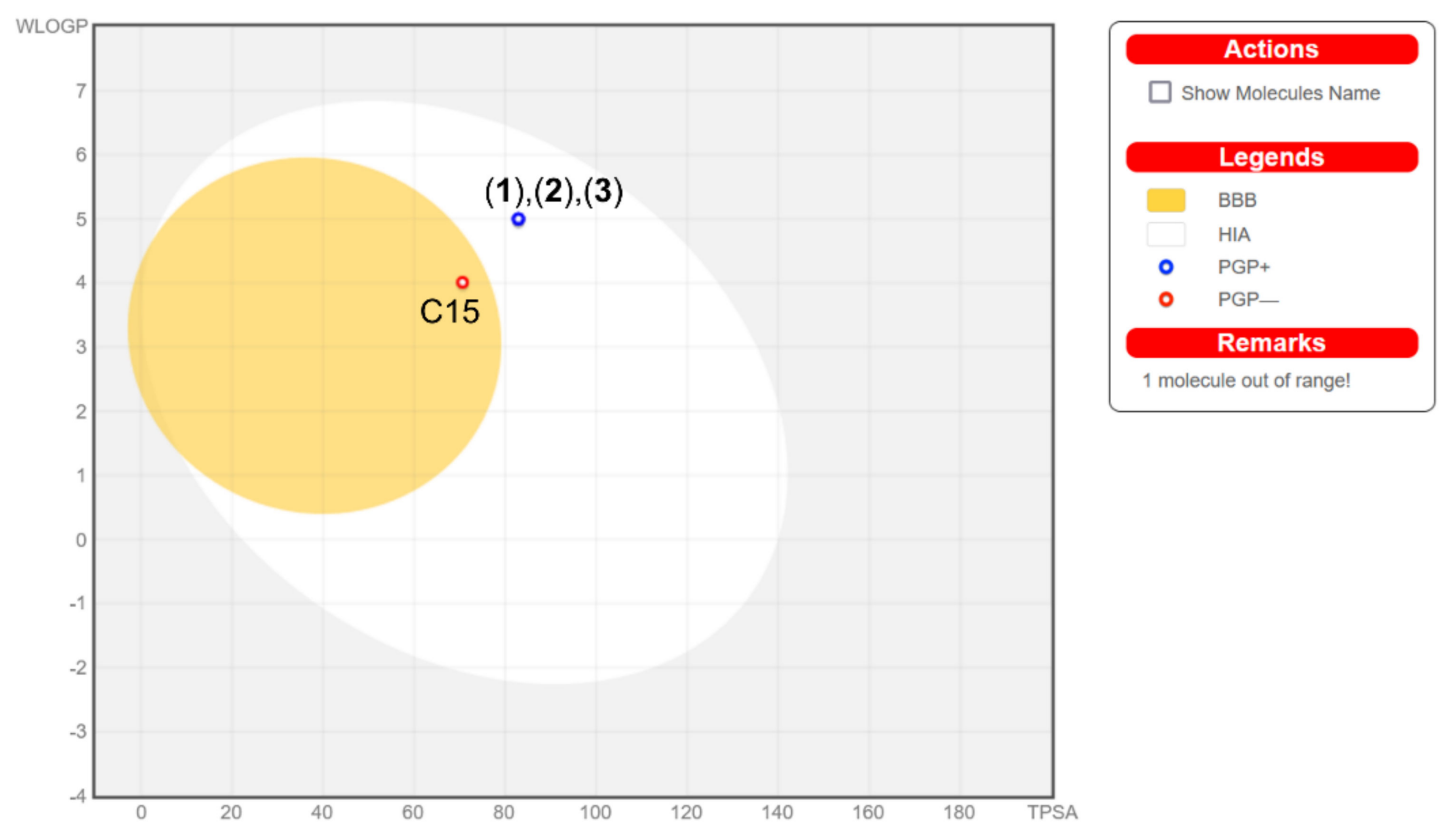
BOILED-Egg diagram for C15 and complexes.

Compound C15 has a high absorption in the gastrointestinal tract, which may make it an effective drug. It is not a substrate of P-gp, which means it is a good candidate against multidrug resistant cancer cells. A bioavailability radar for ligand was made (Figure 7a). For drug-like properties, the compound was found to have a good bioavailability score (0.55) [65] and was consistent with Lipiński’s five principle. This compound satisfies the rules of Lipinski [66], Ghose [67], Egan [68], Veber [69] and Muegge [70]. In the BOILED-Egg diagram (Figure 8), the compound both penetrates the blood-brain barrier and is able to be absorbed gastrointestinally. The ADME parameters indicate that the tested C15 was a good candidate in the search for a biologically active compound. Predicting the putative drug–drug interaction by inhibiting cytochrome P450 (CYP) shows that the compound may be an inhibitor of CYP1A2, CYP2C19, CYP2C9 and CYP3A4, but not CYP2D6. The compound is not available through the skin as indicated by a negative logKp value (−6.0 cm/s). The C15 complexes with Mn(II), Fe(II) and Ni(II) have the same bioavailability radar (Figure 7b). This compound meets with the rules of Lipinski, Ghose, Egan and Veber, (the Ni(II) complex does not meet the Ghose rules). In the BOILED-Egg scheme (Figure 8), the complexes penetrate the gastrointestinal tract. In contrast, the Cu(II) complex (Figure 7c) coordinate two C15 molecules. Both Cu(II) and Zn(II) are too large and do not meet the molecular weight condition. Both complexes do not penetrate the blood-brain barrier and are absorbed through the gastrointestinal tract. Servis ProTox II classified ligand to toxicity class 4 (predicted LD_50_: 600 mg/kg), (**1**) to toxicity class 4 (predicted LD_50_: 1052 mg/kg), (**2**) to toxicity class 2 (predicted LD_50_: 8 mg/kg), (**3**) to toxicity class 4 (predicted LD_50_: 1052 mg/kg), (**4**) to toxicity class 5 (predicted LD_50_: 2500 mg/kg) and (**5**) to toxicity class 2 (predicted LD_50_: 8 mg/kg).

### 2.9. In Vitro Cytotoxicity of Compounds against A549 and HT29 Cancer Cells

**Figure 9:**
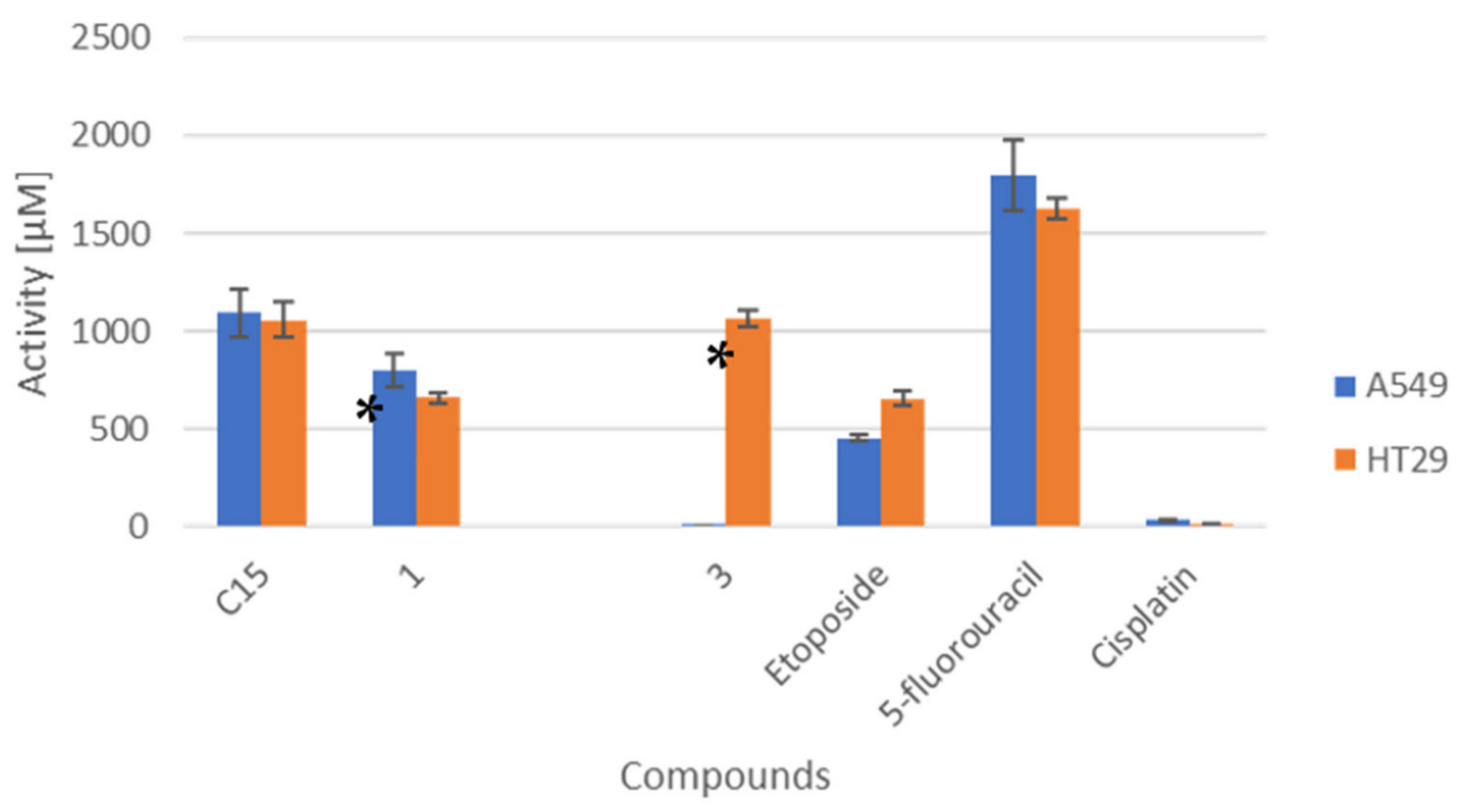
In vitro cytotoxic effect of C15 and C15 complexes on A549 and HT29 cells after 24 h of incubation. Statistical significance was assessed using one-way ANOVA and a post-hoc analysis was performed. * *p* < 0.05 was considered significant for compounds (**1**) and (**3**).

**Figure 10:**
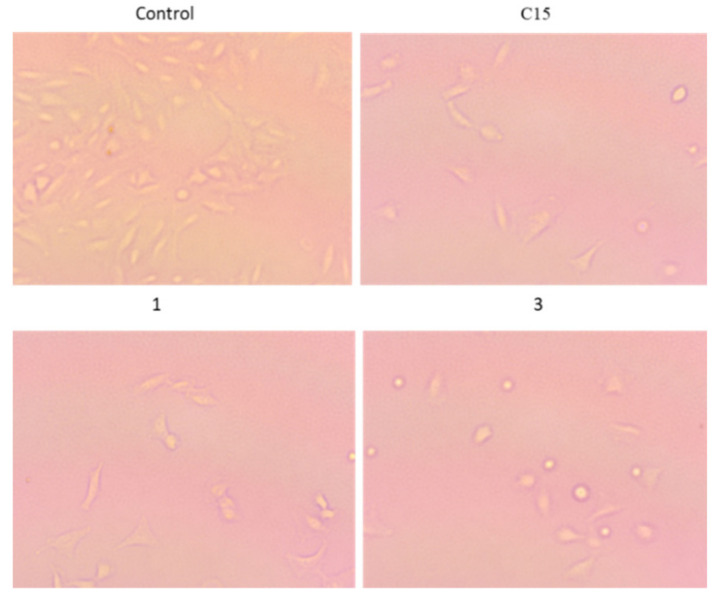
Effect of compounds C15, (**1**) and (**3**) on A549 cell growth. A549 cells were cultured. In the presence or absence (control) of compound. Representative phase-contrast cell images are shown after 24 h in 100× magnification.

The cytotoxic effects of new compounds (C15 and complexes **1**–**5**) were tested against A549 and HT29 cancer cell lines using MTT assay [71,72] Standard anticancer drugs (Etoposide, 5-fluorouracil and Cisplatin) were used as a reference compounds [73,74]. Half of the new complexes (with (**2**), (**4**) and (**5**)) do not show cytotoxicity. When comparing the potency of cytotoxic activity for HT29 cancer cells for the ligand (IC_50_ = 1058.02 ± 87.39 µM) to the complexes, we can see that the compound with (**1**) showed better (IC_50_ = 654.31 ± 25.09 µM) and the compound with (**3**) shows similar activity (IC_50_ = 1064.05 ± 43.56 µM) (Table 3). For the A549 cell line, compound (**1**) also showed better activity to ligand (IC_50_ = 1092.35 ± 124.84 C15 and 794.37 ± 83.62 (complex (**1**)). Compound (**3**) did not show activity in the used range of concentrations. Our present study showed that complexes derivatives containing (**1**) and (**3**) compounds were effective, and the first compound was much more effective in the inhibition of human colon cancer cells growth compared to other metal complexes ((**2**), (**4**) and (**5**)), see Figure 9. Moreover, its potency was comparable with etoposide (IC_50_ = 654.03 ± 39.51). These results clearly suggest that complexes (**1)** and (**3**) were more selective for colon cancer cells than lung cancer cells (Table 3). Additionally, ligand, and metal complexes (**1**) and (**3**) were more potent than 5-fluorouracil. Figure 10 presents the effect of compounds C15, (**1**) and (**3**) on A549 cell growth.

All values are presented as the means ± standard deviation (SD)IC50—50% inhibition of the cell viability, µmol/L (µM).

Statistical significance was assessed using one-way ANOVA and a post-hoc analysis was performed. * p < 0.05 was considered as significantly different between complexes and ligand.

### 2.10. Molecular Docking

**Figure 11:**
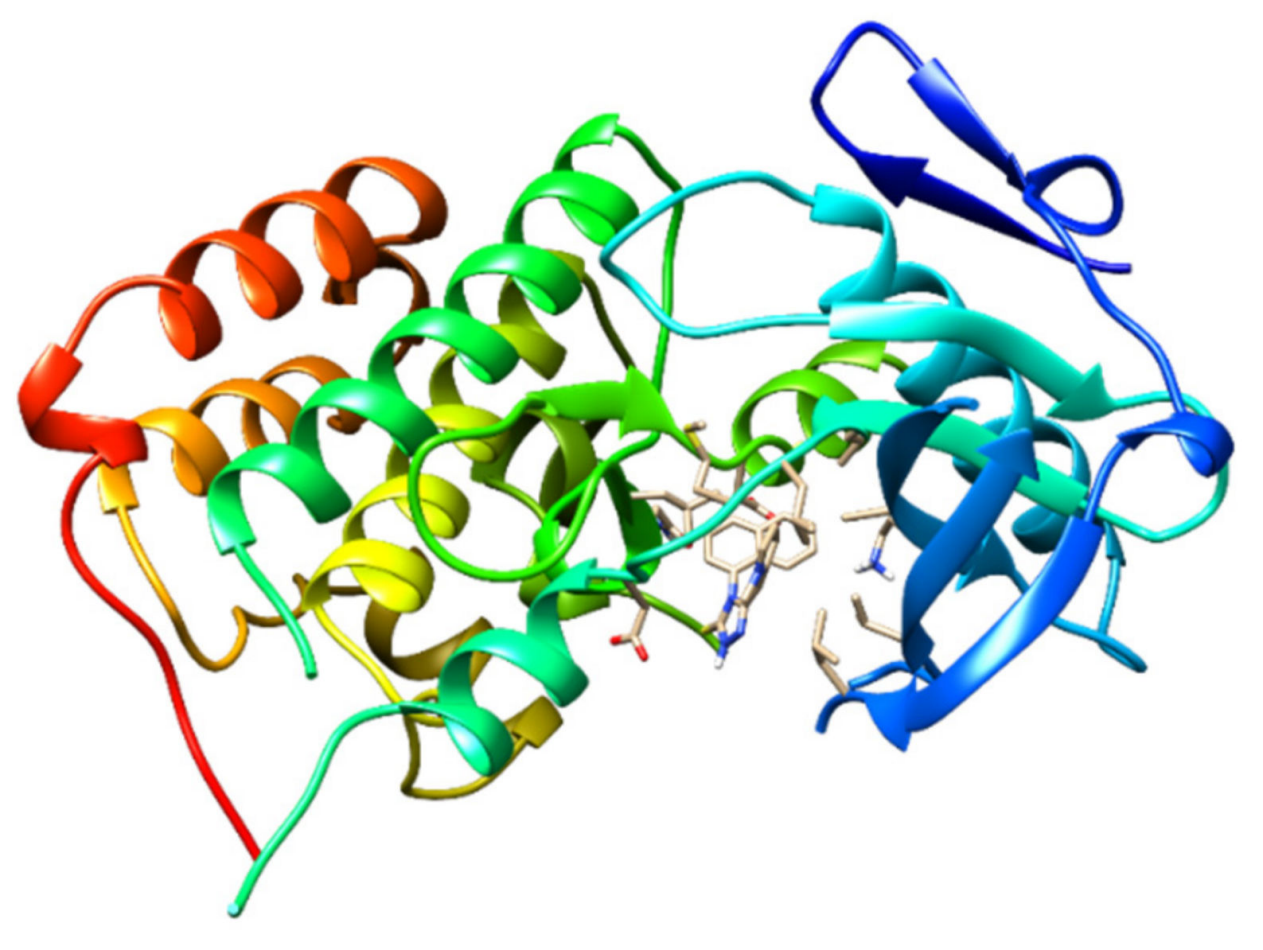
The active sites of the target protein anaplastic lymphoma kinase (PDB ID: 2XP2).

**Figure 12:**
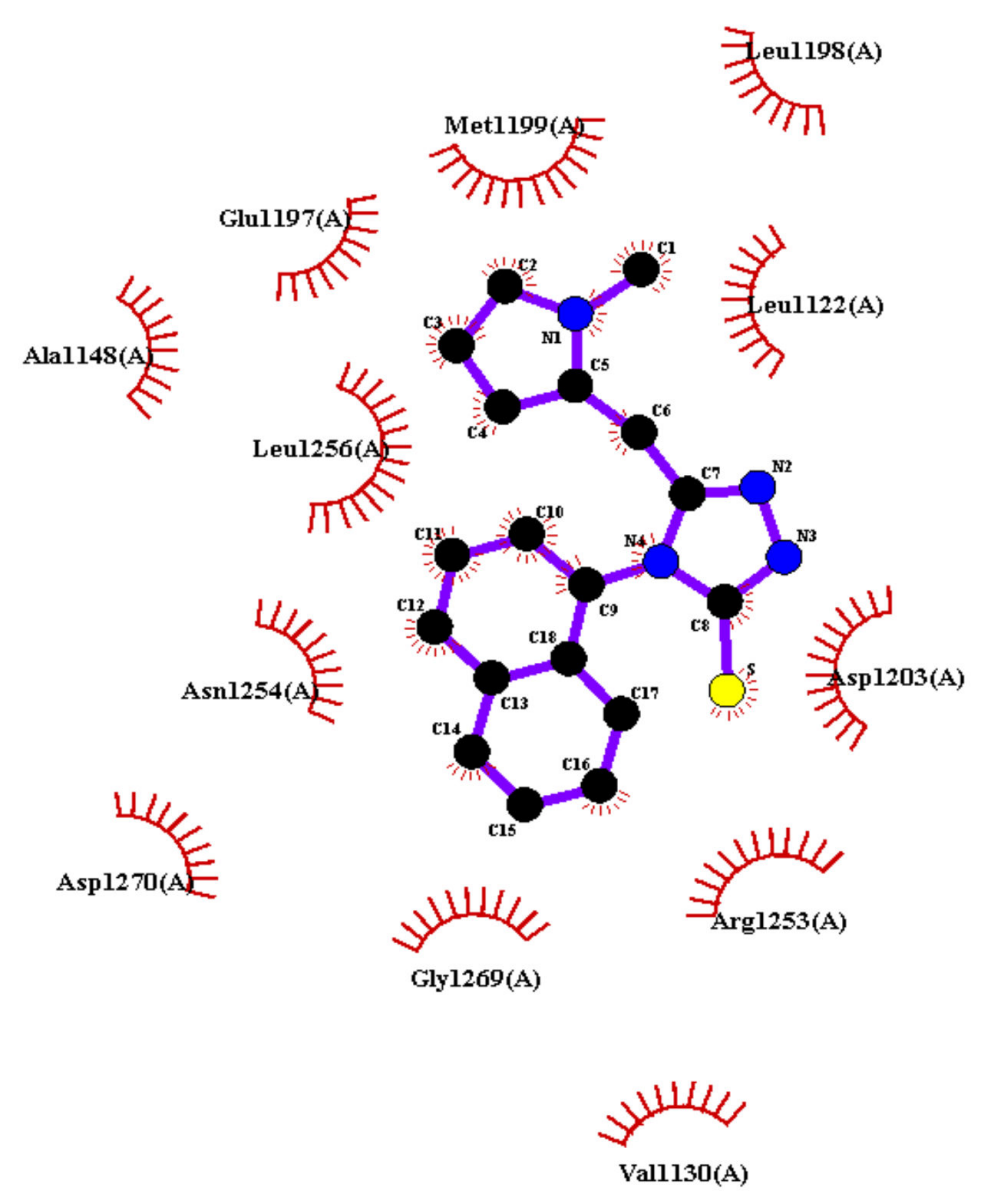
Hydrophobic interactions between titled organic ligand and amino acid residues.

The molecular docking procedure was used to study the interaction of ligands with the model protein system using the GOLD package [75]. The target protein anaplastic lymphoma kinase (PDB ID: 2XP2), which was used in the research of Prabhu Mahendran, was also used in the analysis of compound C15 [76]. This compound binds effectively at the active site. The structure and active site are presented in Figure 11. The results of the molecular docking of ligands are presented in Figure 12. The molecular docking shows a hydrophobic interaction between ligand and protein.

## 3. Materials and Methods

### 3.1. Synthesis of 2-(1-Methylpyrrol-2-yl)Acetohydrazide (A)

Methyl 1-methylpyrrole-2-acetate (1.4 g, 0.01 mol), 5 mL anhydrous ethanol and 0.8 mL (0.02 mol) hydrazine hydrate was heated under reflux condition for 3 h. After cooling, the precipitate was formed, filtered off, dried and crystallized from ethanol. M.p. 112 °C [77].

### 3.2. Synthesis of 1-(1-Methylpyrrol-2-yl)Acetyl-4-(1-Naphtyl) Thiosemicarbazide (B)

The 2-(1-methylpyrrol-2-yl)acetohydrazide (1.53 g, 0.01 mol) was dissolved in methanol (15 mL) and 1-naphtylisothiocyante (1.85 g, 0.01 mol) was added. The mixture was stirred at the reflux temperature of the solvent for 1 h. The mixture was then cooled, and the pure compound was crystallized from the ethanol solution.

Yield: 80%. M.p. 100–112 °C. ^1^H NMR (600 MHz, DMSO-*d*_6_): δ, ppm 3.38 (s, 3H, CH_3_), 3.54 (s, 2H, CH_2_), 5.90–6.53 (m, 3H, CH-pyrrol), 7.50–7.98 (m, 8H, CH-naphtyl), 9.71 (s, 1H, NH), 9.89 (s, 1H, NH), 10.21 (s, 1H, NH). ^13^C NMR (150 MHz, DMSO-*d*_6_): δ, ppm 34, 56, 106, 108, 122, 123, 124, 126, 127, 128, 129, 130, 134, 136, 169, 183 (Figure S1).

### 3.3. Synthesis of 5-((1-Methyl-Pyrrol-2-yl)Methyl)-4-(Naphthalen-1-yl)-1,2,4-Triazoline-3-Thione (C15)

1-(1-Methylpyrrol-2-yl)acetyl-4-(1-naphtyl)thiosemicarbazide (3.3 g, 0.01 mol) and then added 30 mL of 2% NaOH solution. The mixture was then heated to reflux for 2 h. Then the solution was cooled and 3M HCl solution was added. The colour precipitate compound (after drying) was crystallized from ethanol.

Yield 76%, M.p. 248 °C. ^1^H NMR (600 MHz DMSO-*d*_6_): δ, ppm 3.37 (s, 2H, CH_3_), 3.63 (s, 2H, CH_2_), 5.28–6.42 (m, 3H, CH-pyrrol), 7.16–8.14 (m, 8H, CH-naphtyl), 13.95 (s, 1H, NH). FT-IR (KBr): 3095, 3050, 2932, 2778, 1700, 1649, 1597, 1503, 1470, 1435, 1413, 1336, 1308, 1005, 959, 804, 774, 707, 660 cm^−^^1^ (Figure S2).

### 3.4. Synthesis of the Metal(II) Complexes Containing Heterocyclic 4,5-Disbustituted 1,2,4-Triazole-3-Thione (1-5)

The aimed complexes were prepared by mixing the methanolic solutions of appropriate transition metal chlorides [M = Mn(II), Fe(II), Ni(II), Cu(II) and Zn(II)] with 5-((1-methyl-pyrrol-2-yl)methyl)-4-(naphthalen-1-yl)-1,2,4-triazoline-3-thione in equimolar ratio 1:1. A methanol solution of C15 (100 mg, 0.31 mmol) in (25 mL of CH_3_OH) was very slowly heated up to 60 °C to completely dissolve the organic ligand. A solution of appropriate anhydrous metal(II) chloride (100 mg, 0.31 mmol in 15 mL of methanol) was partially added to the ligand solution. The total volume was 70 mL. The reaction mixture was stirred at room temperature for 3 h. The reaction mixture was then stored in the fume hood for two days to observe the solid product’s sedimentation and to carry out the filtration. The solid product obtained was washed methanol used for further characterization.

Mn(C15)Cl_2_MeOH (**1**)**.** Yield: 65%. FT-IR (KBr): 3520, 3503, 3392, 3322, 3083, 3034, 2916, 2762, 2733, 1630, 1610, 1563, 1489, 1403, 1341, 1312, 1289, 1259, 1089, 1007, 753, 714 cm^−1^. Molecular formula (C_19_H_20_OCl_2_MnN_4_S): Calculated: C; 47.71, H; 4.22, N; 11.71, Mn; 11.49, S; 6.70. Found: C; 48.19, H; 3.98, N; 12.14, Mn; 11.81, S; 7.37 (Table S1).

Fe(C15)Cl_2_MeOH (**2**). Yield: 50%. FT-IR (KBr): 3397, 3242, 3094, 3047, 2930, 2777, 2723, 1636, 1613, 1572, 1495, 1412, 1336, 1308, 1088, 1005, 806, 774, 706 cm^−1^. Molecular formula (C_19_H_20_OCl_2_FeN_4_S): Calculated: C; 47.62, H; 4.21, N; 11.69, Fe; 11.65, S; 6.69. Found: C; 47.87, H; 4.00, N; 12.11, Fe; 12.56, S; 6.61 (Table S1).

Ni(C15)Cl_2_MeOH (**3**). Yield: 60%. FT-IR (KBr): 3522, 3506, 3447, 3190, 3093, 2919, 2849, 2777, 2722, 1629, 1603, 1572, 1494, 1413, 1337, 1308, 1088, 1005, 806, 774, 706 cm^−1^. Molecular formula (C_19_H_20_OCl_2_NiN_4_S): Calculated: C; 47.34, H; 4.18, N; 11.62, Ni; 12.19, S; 6.65. Found: C; 47.98, H; 3.96, N; 12.06, Ni; 13.08, S; 7.35 (Table S1).

Cu(C15)_2_Cl_2_ (**4**). Yield: 68%. FT-IR (KBr): 3444, 3055, 3013, 2940, 2845, 1701, 1631, 1596, 1509, 1468, 1436, 1406, 1307, 1185, 958, 803, 772, 661 cm^−1^. Molecular formula (C_36_H_32_Cl_2_CuN_8_S_2_): Calculated: C; 55.77, H; 3.64, N; 14.45, Cu; 8.19, S; 8.27. Found: C; 55.89, H; 3.66, N; 14.61, Cu; 8.52, S; 8.34 (Table S1). 

Zn(C15)_4_Cl_2_ (**5**). Yield: 50%. FT-IR (KBr): 3419, 3092, 3047, 2930, 2777, 2722, 1632, 1572, 1495, 1412, 1337, 1307, 1088, 1004, 958, 806, 774, 705 cm^−1^. Molecular formula (C_72_H_64_Cl_2_ZnN_16_S_4_): Calculated: C; 60.99, H; 3.98, N; 15.80, Zn; 4.61, S; 9.05. Found: C; 60.51, H; 3.96, N; 15.49, Zn; 4.85, S; 8.48 (Table S1).

### 3.5. Chemistry

All chemicals used for the synthesis were purchased from Sigma-Aldrich (St. Louis, MO, USA), Alfa Aesar (Haverhill, MA, USA) and POCH (Gliwice, Poland) companies and used without further purification. Melting points were determined using Fisher–Johns block and Stuart (SMP30) and presented without corrections. The ^1^H and ^l3^C NMR spectra were recorded on a Bruker Avance 600 spectrometer (Bruker BioSpin GmbH, Rheinstetten, Germany) in DMSO-D_6_. Chemical shifts are reported in parts per million (ppm, δ scale) relative either to internal standard (TMS) or residual solvent peak. Anhydrous Mn(II) chloride, anhydrous Fe(II) chloride, anhydrous Ni(II) chloride, anhydrous Cu(II) chloride, and anhydrous Zn(II) chloride were obtained from Sigma-Aldrich, Germany. FT-IR spectra of free organic ligand and metal complexes were recorded with an IR Tracer-100 Schimadzu Spectrometer (4000–600 cm^−1^ with an accuracy of recording of 1 cm^−1^, Kyoto, Japan) using KBr pellets. Magnetization measurements were carried out in a Quantum Design 7T Magnetic property measurement system (MPMS-7T) in the magnetic field range 0 *T* < μ_0 H < 7 T and temperature interval 2 K < *T* < 300 K. The thermal analyses were carried out with a Netzsch Iris 209 thermoanalyzer (Netzsch-Geratebau GmbH, Selb, Germany). Samples were heated in corundum crucibles up to 1000 °C, with a 10 °C/min heating rate, in the atmosphere of synthetic air (20% O_2_, 80% N_2_).

### 3.6. Inductively Coupled Plasma Analysis

The analysis of metal(II) concentration and sulfur were prepared for all coordination compounds. Samples (each of about 20 mg) were digested in the mixture of concentrated acids (1 mL of 36% HCl and 6 mL of 65% HNO_3_) and decomposed using the Anton Paar Multiwave 3000 closed system instrument. Mineralization was carried out for 45 min at 240 °C under 60 bar pressure. The contents of Mn(II), Fe(II), Ni(II), Cu(II), and Zn(II) in the samples were determined by the ICP-OES, Plasma Quant PQ 9000 Elite (Analytik Jena, Jena, Germany). Absorbances were measured at analytical spectral lines: 257.610 nm for Mn(II), 259.940 nm for Fe(II), 231.604 nm for Ni(II), 324,754 nm for Cu(II), 206.200 nm for Zn(II) and 180.672 nm for sulphur. Standard solutions Merck (1000 mg/L) were used for the preparation of calibration curves.

### 3.7. Electron Paramagnetic Resonance Measurement

EPR measurements were carried out at room temperature using Bruker ELEXSYS E580 spectrometer equipped with a TE102 resonance cavity. The spectrometer operated at X-band microwaves with frequencies of approximately 9.38 GHz. Due to small changes in microwave frequencies between individual runs, for better visual comparison, the presented spectra were recalculated to 9.5 GHz. The presented spectra were measured for microwave power 0.47 mW, but the power dependence was measured to make sure that signal was not saturated. To obtain the g-factor values, for all the symmetrical signals, the simple derivative of Lorentz or Gauss profile was fitted, while for the asymmetrical signal, the simulation using the pepper function of the Easyspin Matlab toolbox was applied [https://doi.org/10.1016/j.jmr.2005.08.013 accessed on 5 July 2022].

### 3.8. PXRD Analysis

Powder diffraction experiments were performed using a Panalytical Empyrean powder diffractometer (240 mm goniometer radius) equipped with a sealed copper tube, a Bragg-Brentano geometry and a Pixcel3D sensitive detector. Divergence slit of 1/8° and 0.02 rad. Soller slits (in both incident and diffracted beam paths) were applied. Generator settings used during the experiment were 45 kV and 40 mA, providing an intense incident beam. The powder sample was packed inside a low background Si sample holder mounted on a goniometric head and rotated during a two-scan measurement. The registered data range was 5° to 30° 2θ with a step of 0.0131° at ambient conditions. Obtained scans were tested for discrepancies and summed, since no significant difference was observed.

### 3.9. ADMET Analysis

An ADME analysis was performed using the SwissADME service (Swiss Institute of Bioinformatics 2021): A free web tool to evaluate pharmacokinetics, drug-likeness and the medicinal chemistry friendliness of small molecules [78]. BOILED-Egg was used to Predict Gastrointestinal Absorption and Brain Penetration of Small Molecules [79]. The ProTOX II service was used to predict toxicities for ligand [80].

### 3.10. Cell Culture

Anticancer activities of the C15 and new complexes (**1**–**5**) were tested on A549-human lung and HT29-human colon cells. They were purchased from the European Collection of Cell Cultures (ECACC, Salisburg, UK) and American Type Culture Collection (Manassas, VA, USA). A549 cells were grown in Dulbecco’s Modified Eagle’s Medium (DMEM) (PAN-Biotech, Aidenbach, Germany), 10% Fetal Bovine Serum (Sigma Aldrich, St. Louis, MO, USA), 2 mM Glutamine (Sigma Aldrich), and 100 units/mL penicillin with 100 mg/mL streptomycin (Sigma Aldrich) were used. The HT29 cells were grown in F12K medium (HyClone, Cramlington, UK) with 10% heat-inactivated fetal bovine serum, FBS (Lonza, Basel, Switzerland), 100 units/mL penicillin with 100 mg/mL streptomycin (Lonza). Both cell lines were grown at 37 °C with 5% CO_2_.

### 3.11. Cytotoxicity Assay

The cell viability was quantified by using 3-(4,5-Dimethylthiazol-2-yl)-2,5-diphenyltetrazolium Bromide (MTT) assay. MTT uses the reducing properties of the marker. In this test, cells were seeded into 96-well plates at density 1 × 10^4^ cells per well. Next, it was cultured for 24 h at 37 °C and 5% CO_2_. Then, the medium was removed and 100 μL of the compound solution was added over a range of concentrations. Compounds were dissolved in DMSO, and medium (final DMSO concentration was less than 0.2%). Cells as a black control were prepared in the same way, but culture medium without compound and pure DMSO was used as a control. Next, plates with compound solutions were incubated for 24 h. After 24 h, medium was removed and MTT solution (50μL) was added, and incubated for 2 h at 37 °C. Later, solution was carefully removed, and 100 μL of DMSO was added to each well. After 10 min at room temperature, the plate was mixed and the absorbance was measured at a wavelength of 570 nm (Synergy H1, BioTek, Winooski, VT, USA). The cell viability was expressed as a IC_50_ [81,82].

## 4. Conclusions

This work describes the synthesis of 5-((1-methyl-pyrrol-2-yl)methyl)-4-(naphthalen-1-yl)-1,2,4-triazoline-3-thione and its five new solid coordination compounds. All the compounds synthesized for the first time were characterized by various analytical and spectroscopic methods. Based on diffraction patterns, it was found that the (**1**) and (**3**) complexes are isostructural. Among all five complexes, the complex (**1**) showed the best cytotoxicity activity against both colon (IC50 = 654.31* ± 25.09 μM) and lung (IC50 = 794.37*± 83.62 μM) cancer lines, and was comparable with the free organic ligand and commercially available drugs in the market such as 5-fluorouracil and Etoposide. The compound (**3**) also exhibited better cytotoxicity activity against colon cancer cells than the other compounds. This is a significant achievement that clearly indicates that more research on the mechanism of their binding to a particular target—active site (enzyme, protein), and their potential use as a cytostatic drug is needed. For these two coordination compounds, potential use as anticancer drugs for the two most common types of cancers, lung and colon cancers, requires a great deal of additional research.

## Figures and Tables

**Table 1:** The g-factors obtained from EPR measurements and the line profile.

Complex	g-Factor	Profile
(**1**)	g_iso_ = 2.021	Gauss
(**2**)	g_iso_ = 2.016	Gauss
(**3**)	g_iso_ = 2.26	Gauss
(**4**)	[*g*_⊥_, *g*_||_] = [2.05, 2.24]	Gauss
(**5**)	g_iso_ = 2.003	Lorentz

**Table 2:** Cancer cell line prediction results of C15.

Pa	Pi	Cell-Line Name	Tissue/Organ
0.526	0.004	Pancreatic carcinoma	Pancreas
0.518	0.018	Renal carcinoma	Kidney
0.365	0.007	Urothelial bladder carcinoma	Urinary tract
0.362	0.061	Colon carcinoma	Colon
0.295	0.084	Leukemic T-cells	Blood
0.289	0.088	Adult immunoblastic lymphoma	Haematopoietic and lymphoid tissue
0.188	0.004	Bladder carcinoma	Urinary tract
0.297	0.125	Glioblastoma	Brain
0.282	0.139	Melanoma	Skin
0.250	0.156	Non-small cell lung carcinoma	Lung
0.238	0.146	Non-small cell lung carcinoma	Lung
0.229	0.153	Colon adenocarcinoma	Colon

Pa (probability “to be active”). Pi (probability “to be inactive”).

**Table 3:** In vitro cytotoxic activity of new compounds (**1**)–(**5**) and C15 on two cancer cell lines (A549 and HT29).

Compound	IC_50_ against A549 (µM)	IC_50_ against HT29 (µM)
C15	1092.35 ± 124.84	1058.02 ± 87.39
(**1**)	794.37 ± 83.62	654.31 ± 25.09
(**2**)	no activity	no active
(**3**)	no activity	1064.05 ± 43.56
(**4**)	no activity	no activity
(**5**)	no activity	no activity
Etoposide	451.47 ± 18.27	654.03 ± 39.51
5-fluorouracil	>1800	1626.85 ± 49.26
Cisplatin	31.25 ± 1.76 [74]	11.9 ± 4.1 [75]

## Data Availability

Not applicable.

## Supplementary Materials

The following supporting information can be downloaded at: https://www.mdpi.com/article/10.3390/ijms23169162/s1.

## References

[1] Ferlay J., Soerjomataram I., Dikshit R., Eser S., Mathers C., Rebelo M., Parkin D.M., Forman D., Bray F. (2015). Cancer Incidence and Mortality Worldwide: Sources, Methods and Major Patterns in GLOBOCAN 2012. Int. J. Cancer.

[2] Torre L.A., Bray F., Siegel R.L., Ferlay J., Lortet-Tieulent J., Jemal A. (2015). Global cancer statistics, 2012. CA. Cancer J. Clin..

[3] Bray F., Ferlay J., Soerjomataram I., Siegel R.L., Torre L.A., Jemal A. (2018). Global cancer statistics 2018: GLOBOCAN estimates of incidence and mortality worldwide for 36 cancers in 185 countries. CA. Cancer J. Clin..

[4] DeVita V.T., Chu E. (2008). A History of Cancer Chemotherapy. Cancer Res..

[5] Hambley T.W. (2009). Is anticancer drug development heading in the right direction?. Cancer Res..

[6] Neidle S., Thurston D.E. (2005). Chemical approaches to the discovery and development of cancer therapies. Nat. Rev. Cancer.

[7] Orvig C., Abrams M.J. (1999). Medicinal inorganic chemistry: Introduction. Chem. Rev..

[8] Yaman M., Kaya G., Yekeler H. (2007). Distribution of trace metal concentrations in paired cancerous and non-cancerous human stomach tissues. World J. Gastroenterol..

[9] Thompson K.H., Orvig C. (2003). Boon and bane of metal ions in medicine. Science.

[10] Pitucha M., Korga-Plewko A., Czylkowska A., Rogalewicz B., Drozd M., Iwan M., Kubik J., Humeniuk E., Adamczuk G., Karczmarzyk Z. (2021). Influence of complexation of thiosemicarbazone derivatives with Cu (II) ions on their antitumor activity against melanoma cells. Int. J. Mol. Sci..

[11] Raducka A., Czylkowska A., Gobis K., Czarnecka K., Szymański P., Świątkowski M. (2021). Characterization of Metal-Bound Benzimidazole Derivatives, Effects on Tumor Cells of Lung Cancer. Materials.

[12] Raducka A., Świątkowski M., Korona-Głowniak I., Kaproń B., Plech T., Szczesio M., Gobis K., Szynkowska-Jóźwik MI Czylkowska A. (2022). Zinc Coordination Compounds with Benzimidazole Derivatives: Synthesis, Structure, Antimicrobial Activity and Potential Anticancer Application. Int. J. Mol. Sci..

[13] Murugesh N., Haribabu J., Arumugam K., Balachandran C., Swaathy R., Aoki S., Sreekanth A., Karvembu R., Vedachalam S. (2019). NHC-catalyzed green synthesis of functionalized chromones: DFT mechanistic insights and: In vitro activities in cancer cells. New J. Chem..

[14] Subhashree G.R., Haribabu J., Saranya S., Yuvaraj P., Anantha Krishnan D., Karvembu R., Gayathri D. (2017). In vitro antioxidant, antiinflammatory and in silico molecular docking studies of thiosemicarbazones. J. Mol. Struct..

[15] Kalaiyarasi A., Haribabu J., Gayathri D., Gomathi K., Bhuvanesh N.S.P., Karvembu R., Biju V.M. (2019). Chemosensing, molecular docking and antioxidant studies of 8-aminoquinoline appended acylthiourea derivatives. J. Mol. Struct..

[16] Kumar P., Udupi R., Dubey P. (2009). Synthesis and biological study of substituted 1,3,4-oxadiazoles and 1,2,4-triazoles. Int. J. Pharm. Tech Res..

[17] Kaur R., Ranjan Dwivedi A., Kumar B., Kumar V. (2016). Recent developments on 1, 2, 4-triazole nucleus in anticancer compounds: A review. Anticancer Agents Med. Chem..

[18] Ivolgina V.A., Chernov’yants M.S., Popov L.D., Suslonov V.V., Avtushenko N.A., Luanguzov N.V. (2020). Structural study and thermal behavior of novel interaction product of 4-amino-5-(furan-2-yl)-4H-1, 2, 4-triazole-3-thione with molecular iodine. Phosphorus Sulfur Silicon Relat. Elem..

[19] Aggarwal R., Sumran G. (2020). An insight on medicinal attributes of 1, 2, 4-triazoles. Eur J. Med. Chem..

[20] Collin X., Sauleau A., Coulon J. (2003). 1, 2, 4-Triazolo mercapto and aminonitriles as potent antifungal agents. Bioorganic Med. Chem. Lett..

[21] Pibiri I., Buscemi S. (2010). A recent portrait of bioactive triazoles. Curr. Bioact. Compd..

[22] Hemdan M.M., El-Sayed A.A. (2015). Synthesis of Some New Heterocycles Derived from Novel 2-(1,3-Dioxisoindolin-2-yl)Benzoyl Isothiocyanate. J. Heterocycl. Chem..

[23] Hemdan M.M., Fahmy A.F., Ali N.F., Hegazi E., Abd-Elhaleem A. (2008). Synthesis of some new heterocycles derived from phenylacetyl isothiocyanate. Chin. J. Chem..

[24] Hemdan M.M., Elshahawi M.M. (2009). ChemInform Abstract: Synthesis of 1,2,4-Triazoles, Imidazoles, Pyrimidines, Quinazolines, 1,3,5-Triazines, and 1,3-Thiazines from 3-Oxo-5,6-diphenyl-2,3-dihydropyridazine-4-carbonyl Isothiocyanate (I). J. Chem. Res..

[25] Hemdan M.M., El-Sayed A.A.-E., Hemdan M.M., El-Sayed A.A.-E. (2016). Use of Phthalimidoacetyl isothiocyanate as a scaffold in the synthesis of target heterocyclic systems, and their antimicrobial assessment. Chem. Pharm. Bull..

[26] Hemdan M.M. (2009). Addition–cyclisation of 3-(2-thienyl)acryloyl isothiocyanate with hydrazine derivatives as a source of triazoles and thiadiazoles. J. Chem. Res..

[27] Hemdan M.M. (2010). Synthesis and antimicrobial activities of some heterocyclic systems from 2-furoyl isothiocyanate. Phosphorus Sulfur Silicon Relat. Elem..

[28] Hemdan M.M., Fahmy A.F.M., Aly N.F., Hegazi I.A., El-Sayed A.A. (2012). Utility of phthalimidoacyl isothiocyanate in synthesis of quinazolines, benzoxazoles, benzimidazoles, 1, 2, 4-triazoles, and oxatriazepines. Phosphorus Sulfur Silicon Relat Elem..

[29] Gomtsyan A. (2012). Heterocycles in drugs and drug discovery. Chem. Heterocycl. Compd..

[30] Sachs G., Shin J.M., Howden C.W. (2006). Review article: The clinical pharmacology of proton pump inhibitors. Aliment. Pharmacol. Ther..

[31] Tkach V.V., Kushnir M.V., de Oliveira Sílvio C., Shevchenko I.M., Odyntsova V.M., Omelyanchik V.M., Luganska O.V., Kopiika V.V., Kormosh Z.O., Ivanushko Y.G. (2022). Theoretical Description for Anti-COVID-19 Drug Molnupiravir Electrochemical Determination over the Poly-((1,2,4-triazole)-co-(squaraine dye)) Composite with Cobalt (III) Oxyhydroxide. Biointerface Res. Appl. Chem..

[32] El Ashry E.S.H., Farahat Mohamed M.K., Awad Laila F., Mahmoud B., Hoda Y., Badawy Mohamed E.I., Abd Al Moaty Mohamed N.J. (2022). Synthesis, antibacterial, antioxidant, and molecular docking studies of 6-methylpyrimidin-4(3H)-one and oxo-1,2,4-triazolo [4,3-a]pyrimidine derivatives. Mol. Struct..

[33] Alsaad H., Kubba A., Lubna H., Tahtamouni A., Hamzah H. (2022). Synthesis, docking study, and structure activity relationship of novel anti-tumor 1, 2, 4 triazole derivatives incorporating 2-(2, 3-dimethyl aminobenzoic acid) moiety. Pharmacia.

[34] Demirbas A., Sahin D., Demirbas N., Karaoglu S.A. (2009). Synthesis of some new 1,3,4-thiadiazol-2-ylmethyl-1,2,4-triazole derivatives and investigation of their antimicrobial activities. Eur. J. Med. Chem..

[35] Pal D., Singh V., Pandey D.D., Maurya R.K. (2014). Synthesis, characterization and antimicrobial Evaluation of some 1, 2, 4-triazole derivatives. Int. J. Pharm. Pharm. Sci..

[36] Kazeminejad Z., Marzi M., Shiroudi A., Kouhpayeh S.A., Farjam M., Zarenezhad E. (2022). Novel 1, 2, 4-Triazoles as Antifungal Agents. BioMed Res. Int..

[37] Al-Mansury S., Aboktifa M.A., Jassim A.M., Balakit A.A., Alkazazz F.F. (2022). Evaluation the Antioxidant Enzymes Activity in Adults Male Rats Treated with Some New 3-mercapto1, 2, 4-triazole Derivatives. Res. J. Pharm. Technol..

[38] Li J., Zhang J. (2022). The Antibacterial Activity of 1, 2, 3-triazole-and 1, 2, 4-Triazole-containing Hybrids against Staphylococcus aureus: An Updated Review (2020-Present). Curr. Top. Med. Chem..

[39] Hou Y.P., Sun J., Pang Z.H., Lv P.C., Li D.D., Yan L., Zhang H.J., Zheng E.X., Zhao J., Zhu H.L. (2011). Synthesis and antitumor activity of 1,2,4-triazoles having 1,4-benzodioxan fragment as a novel class of potent methionine aminopeptidase type II inhibitors. Bioorganic Med. Chem..

[40] Lin R., Connolly P.J., Huang S., Wetter S.K., Lu Y., Murray W.V., Emanuel S.L., Gruninger R.H., Fuentes-Pesquera A.R., Rugg C.A. (2005). 1-Acyl-1H-[1,2,4]triazole-3,5-diamine analogues as novel and potent anticancer cyclin-dependent kinase inhibitors: Synthesis and evaluation of biological activities. J. Med. Chem..

[41] Saad H.A., Osman N.A., Moustafa A.H. (2011). Synthesis and analgesic activity of some new pyrazoles and triazoles bearing a 6,8-dibromo-2-methylquinazoline moiety. Molecules.

[42] Kumidha J.T.D., Leonard M., Muthumani N., Chidhambaranathan T.K. (2013). Synthesis and evaluation of some 1, 2, 4-triazole derivatives as anticonvulsant, anti-inflammatory and antimicrobial agents. Asian J. Pharm. Clin..

[43] Shashikala M.D.K., Ramaiah G.K., Vanita K., Veena V.P.V. (2011). Synthesis and analgesic activity of triazolothiadiazoles and triazolothiadiazines encompassing 3–nitronaphtho[2,1-b]furan. Chem. Pharm. Res..

[44] Murty M.S.R., Ram K.R., Rao B.R., Rao R.V., Katiki M.R., Rao J.V., Pamanji R., Velatooru L.R. (2014). Synthesis, characterization, and anticancer studies of S and N alkyl piperazine-substituted positional isomers of 1,2,4-triazole derivatives. Med. Chem. Res..

[45] Cao X., Wang W., Wang S., Bao L. (2017). Asymmetric synthesis of novel triazole derivatives and their in vitro antiviral activity and mechanism of action. Eur. J. Med. Chem..

[46] Singh R., Kashaw S.K., Mishra V.K., Mishra M., Rajoriya V., Kashaw V. (2018). Design and synthesis of new bioactive 1,2,4-triazoles, potential antitubercular and antimicrobial agents. Indian J. Pharm. Sci..

[47] Gao F., Wang T., Xiao J., Huang G. (2019). Antibacterial activity study of 1,2,4-triazole derivatives. Eur. J. Med. Chem..

[48] Keivanloo A., Fakharian M., Sepehri S. (2020). 1,2,3-Triazoles based 3-substituted 2-thioquinoxalines: Synthesis, anti-bacterial activities, and molecular docking studies. J. Mol. Struct..

[49] Haegler P., Joerin L., Krähenbühl S., Bouitbir J. (2017). Hepatocellular toxicity of imidazole and triazole antimycotic agents. Toxicol. Sci..

[50] Foroumadi A., Mansouri S., Kiani Z., Rahmani A. (2003). Synthesis and in vitro antibacterial evaluation of N-[5-(5-nitro-2-thienyl)-1,3,4-thiadiazole-2-yl] piperazinyl quinolones. Eur. J. Med. Chem..

[51] Zmejkovski B.B., Savić A., Poljarević J., Pantelić N., Aranđelović S., Radulović S., Sabo T.J. (2014). Synthesis, characterization and in vitro antitumor activity of new palladium (II) complexes with (S, S)-R2edda-type esters. Polyhedron.

[52] Aguilà D., Escribano E., Speed S., Talancón D., Yermán L., Alvarez S. (2009). Calibrating the coordination chemistry tool chest: Metrics of bi-and tridentate ligands. Dalton Trans..

[53] Gavrilova A.L., Bosnich B. (2004). Principles of mononucleating and binucleating ligand design. Chem. Rev..

[54] Frezza M., Hindo S., Chen D., Davenport A., Schmitt S., Tomco D., Ping Dou Q. (2010). Novel metals and metal complexes as platforms for cancer therapy. Curr. Pharm. Des..

[55] Kozyra P., Korga-Plewko A., Karczmarzyk Z., Hawrył A., Wysocki W., Człapski M., Iwan M., Ostrowska-Leśko M., Fornal E., Pitucha M. (2022). Potential Anticancer Agents against Melanoma Cells Based on an As-Synthesized Thiosemicarbazide Derivative. Biomolecules.

[56] Pitucha M., Korga-Plewko A., Kozyra P., Iwan M., Kaczor A.A. (2020). 2, 4-Dichlorophenoxyacetic Thiosemicarbazides as a new class of compounds against stomach cancer potentially intercalating with DNA. Biomolecules.

[57] Bharty M.K., Bharti A., Chaurasia R., Chaudhari U.K., Kushawaha S.K., Sonkar P.K., Ganesan V., Butcher J.R. (2019). Synthesis and characterization of Mn (II) complexes of 4-phenyl (phenyl-acetyl)-3-thiosemicarbazide, 4-amino-5-phenyl-1, 2, 4-triazole-3-thiolate, and their application towards electrochemical oxygen reduction reaction. Polyhedron.

[58] Paqhaleh D.M.S., Aminjanov A.A., Amani V.J. (2014). Discrete and polymeric lead (II) complexes containing 4-methyl-1, 2, 4-triazole-3-thiol ligand: X-ray studies, spectroscopic characterization, and thermal analyses. Inorg. Organomet. Polym..

[59] Kahn O. (2015). Introduction to Molecular Magnetism.

[60] Sivasankar Reddy M., Prathima B., Saraswathi M., Babu S., Sarala Y., Varada Reddy A.J. (2016). Synthesis, spectral aspects and biological activities of 5-hydroxy-2- nitrobenzaldehydethiosemicarbazone and their Mn(II), Co(II) and Ni(II) complexes. Appl. Pharm. Sci..

[61] Hathaway B.J., Billing D.E. (1970). The electronic properties and stereochemistry of mono-nuclear complexes of the copper(II) ion. Coord. Chem. Rev..

[62] Vlček A.J. (2001). Electronic structure of Bis(o-iminobenzo-semiquinonato)metal complexes (Cu, Ni, Pd): The art of establishing physical oxidation states in transition-metal complexes containing radical ligands. Chemtracts.

[63] Rahman K.N.A., Haribabu J., Balachandran C., Bhuvanesh N.S.P., Karvembu R., Sreekanth A. (2017). Copper, nickel and zinc complexes of 3-acetyl coumarin thiosemicarbazone: Synthesis, characterization and in vitro evaluation of cy-totoxicity and DNA/protein binding properties. Polyhedron.

[64] Haribabu J., Alajrawy O.I., Jeyalakshmi K., Balachandran C., Krishnan D.A., Bhuvanesh N., Aoki S., Natarajan K., Karvembu R. (2021). N-substitution in isatin thiosemicarbazones decides nuclearity of Cu(II) complexes—Spectroscopic, molecular docking and cytotoxic studies. Spectrochim. Acta Part A Mol. Biomol. Spectrosc..

[65] Yvonne C., Martin A. (2005). Bioavailability Score. J. Med. Chem..

[66] Lipinski C.A., Lombardo F., Dominy B.W., Feeney P.J. (2012). Experimental and computational approaches to estimate solubility and permeability in drug discovery and development settings. Adv. Drug Deliv. Rev..

[67] Ghose A.K., Viswanadhan V.N., Wendoloski J.J. (1999). A knowledge-based approach in designing combinatorial or medicinal chemistry libraries for drug discovery. 1. A qualitative and quantitative characterization of known drug databases. J. Comb. Chem..

[68] Egan W.J., Merz K.M., Baldwin J.J. (2000). Prediction of Drug Absorption Using Multivariate Statistics. J. Med. Chem..

[69] Veber D.F., Johnson S.R., Cheng H.Y., Smith B.R., Ward K.W., Kopple K.D. (2002). Molecular properties that influence the oral bioavailability of drug candidates. J. Med. Chem..

[70] Muegge I., Heald S.L., Brittelli D.J. (2001). Simple selection criteria for drug-like chemical matter. J. Med. Chem..

[71] Haribabu J., Srividya S., Mahendiran D., Gayathri D., Venkatramu V., Bhuvanesh N., Karvembu R. (2020). Synthesis of palladium (II) complexes via Michael addition: Antiproliferative effects through ROS-mediated mitochondrial apoptosis and docking with SARS-CoV-2. Inorg. Chem..

[72] Haribabu J., Tamura Y., Yokoi K., Balachandran C., Umezawa M., Tsuchiya K., Yamada Y., Karvembu R. (2021). Synthesis and Anticancer Properties of Bis- and Mono(cationic peptide) Hybrids of Cyclometalated Iridium(III) Complexes: Effect of the Number of Peptide Units on Anticancer Activity. Eur. J. Inorg. Chem..

[73] Kaplan A., Akalin C.G., Kutlu M.P. (2017). Titanyum Dioksitin A549 Hücreleri Üzerindeki Apoptotik Etkileri. Tumor Biol..

[74] Lovejoy K.A., Serova M., Bieche I., Emami S., D’Incalci M., Broggini M., Erba E., Gespach C., Cvitkovic E., Faivre S. (2011). Spectrum of Cellular Responses to Pyriplatin, a Monofunctional Cationic Antineoplastic Platinum(II) Compound, in Human Cancer Cells. Mol. Cancer Ter..

[75] Jones G., Willett P., Glen R.C., Leach A.R., Taylor R.J. (1997). Development and validation of a genetic algorithm for flexible docking. Mol. Biol..

[76] Prabhu M., Jeya R.A., Balachandran C., Stalin A., Saravanan R., Loganathan K., Chennakesava R.K., Suresh A., Hiteshkumar B.N. (2018). Synthesis of novel β-amino alcohols from phenylacetylcarbinol: Cytotoxicity activity against A549 cells and molecular docking. Res. Chem. Intermed..

[77] Pitucha M., Wujec M.D. (2004). Synthesis of new derivatives of 3- (1-methylpyrrole-2-ylmethyl) -4-substituted-1,2,4-triazolin-5-thione. Ann. UMCS Lublin..

[78] Daina A., Michielin O., Zoete V. (2017). SwissADME: A free web tool to evaluate pharmacokinetics, drug-likeness and medicinal chemistry friendliness of small molecules. Sci. Rep..

[79] Daina A., Zoete V. (2016). A boiled-egg to predict gastrointestinal absorption and brain penetration of small molecules. ChemMedChem.

[80] Banerjee P., Eckert A.O., Schrey A.K., Preissner R. (2018). ProTox-II: A webserver for the prediction of toxicity of chemicals. Nucleic Acids Res..

[81] Kłosiński K., Girek M., Czarnecka K., Pasieka Z., Skibiński R., Szymański P. (2020). Biological assessment of new tetrahydroacridine derivatives with fluorobenzoic moiety in vitro on A549 and HT-29 cell lines and in vivo on animal model. Hum. Cell.

[82] Girek M., Kłosiński K., Grobelski B., Pizzimenti S., Cucci M.A., Daga M., Barrera G., Pasieka Z., Czarnecka K., Szymański P. (2019). Novel tetrahydroacridine derivatives with iodobenzoic moieties induce G0/G1 cell cycle arrest and apoptosis in A549 non-small lung cancer and HT-29 colorectal cancer cells. Mol. Cell. Biochem..

