# Supplementary material for: New Derivatives of 5-((1-Methyl-Pyrrol-2-yl) Methyl)-4-(Naphthalen-1-yl)-1,2,4-Triazoline-3-Thione and Its Coordination Compounds with Anticancer Activity
Source: Int J Mol Sci. 2022 Aug 15;23(16):9162. doi: 10.3390/ijms23169162 (PMC9409456; doi:10.3390/ijms23169162)
Supplement: Supplementary file 1 [file ijms-23-09162-s001.zip › ijms-1828958-supplementary.pdf]

International Journal of
Molecular Sciences

MDPI

New Derivatives of 5-((1-methyl-pyrrol-2-yl) methyl)-4-(naphthalen-1-yl)-1,2,4-triazoline-3-thione and its Coordination Compounds with Anticancer Activity

Agnieszka Czylkowska [1,*], Suneel Lanka [1], Małgorzata Szczesio [1], Kamila Czarnecka [2], Paweł Szymański [2,3], Monika Pitucha [4], Aneta Drabińska [5], Bruno Cury Camargo [6] and Jacek Szczytko [6]

1 Institute of General and Ecological Chemistry, Faculty of Chemistry, Lodz University of Technology, Zeromskiego 116, 90-924 Lodz, Poland[2] Department of Pharmaceutical Chemistry, Drug Analyses and Radiopharmacy, Faculty of Pharmacy, Medical University of Lodz, Muszynskiego 1, 90-151 Lodz, Poland
3 Department of Radiobiology and Radiation Protection, Military Institute of Hygiene and Epidemiology, 4 Kozielska St., 01-163 Warsaw, Poland
4 Independent Radiopharmacy Unit, Faculty of Pharmacy, Medical University of Lublin, Chodzki 4A, 20-093 Lublin, Poland
5 Faculty of Physics, University of Warsaw, Pasteura 5, 02-093 Warszawa, Poland
6 Institute of Experimental Physics, Faculty of Physics, University of Warsaw, Pasteura 5, 02-093 Warszawa, Poland
* Correspondence: agnieszka.czylkowska@p.lodz.pl

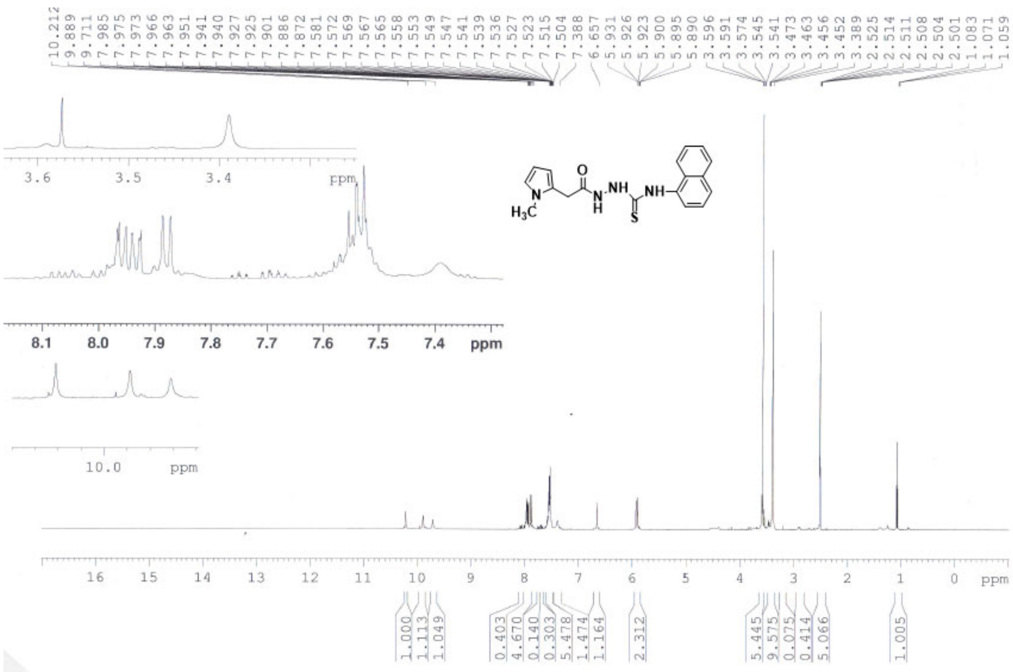

^{1}H-NMR Spectra of Compound B

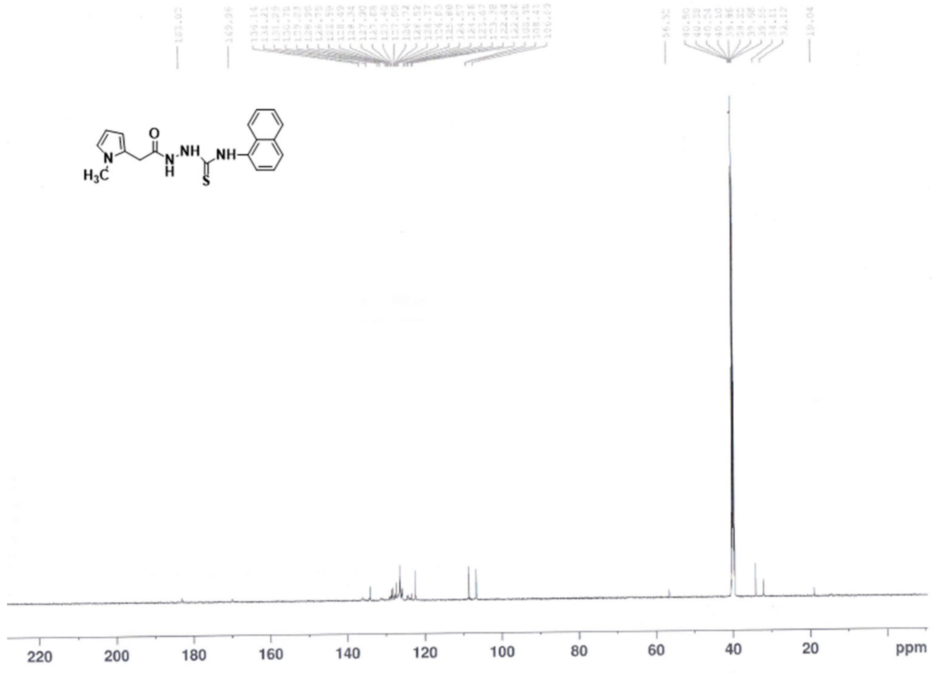

^{13}C-NMR Spectra of Compound B

Figure S1. ^{1}H-NMR and ^{13}C-NMR Spectra of Compound B.

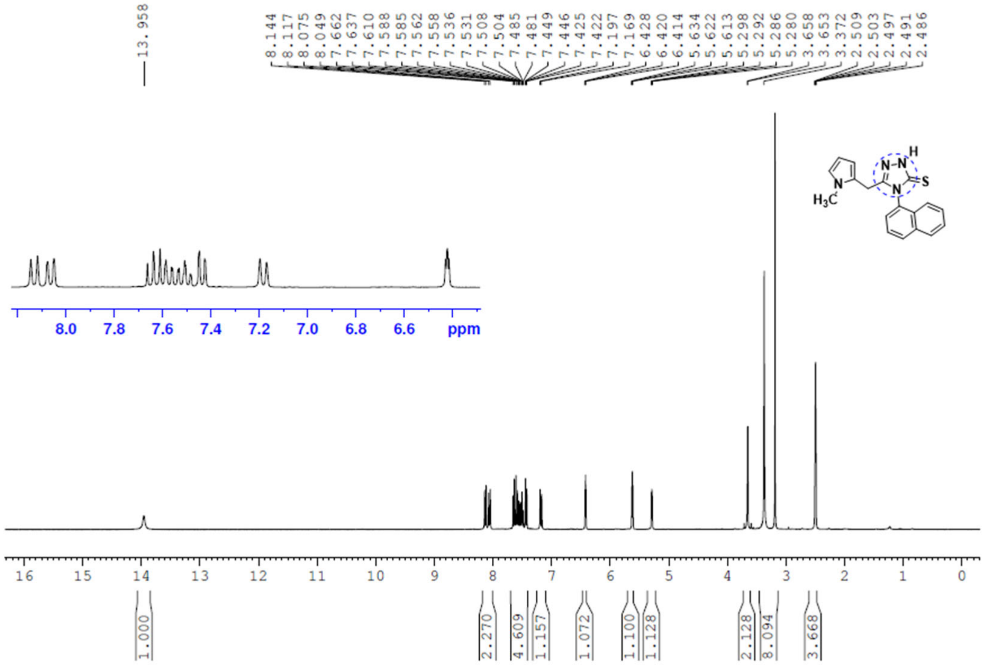

^{1}H-NMR Spectra of Compound C15

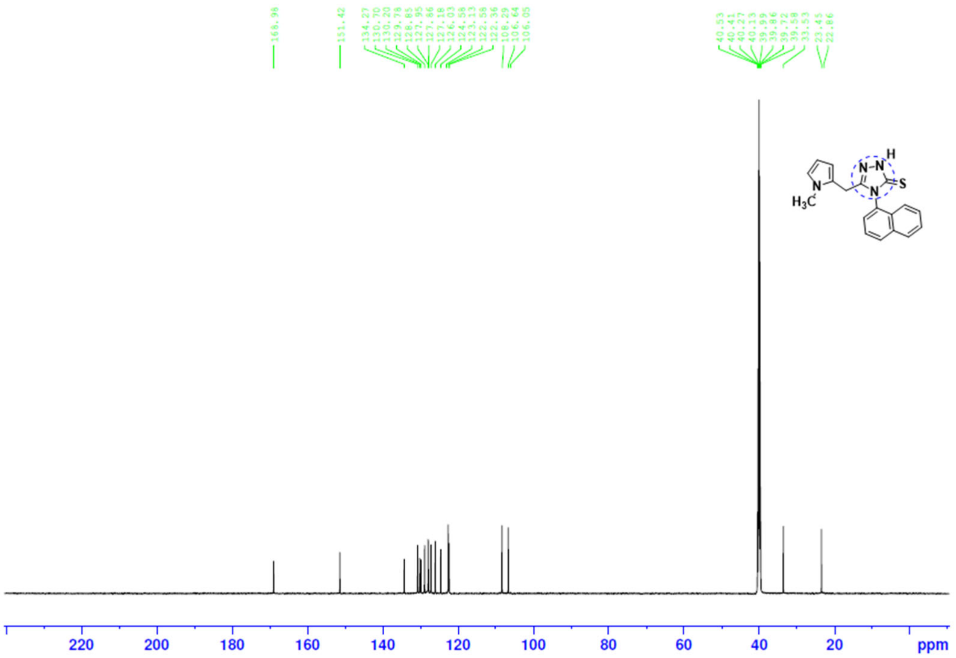

^{13}C-NMR Spectra of Compound C15

Figure S2. ^{1}H-NMR and ^{13}C-NMR Spectra of Compound C15.

Table S1. ICP and CHN analysis of five (**1-5**) new coordination compounds.

No	Complex	M.W (g/mol)	Colour	Metal%		Sulphur%		Carbon%		Hydrogen%		Nitrogen%	
				Calc.	Found	Calc.	Found	Calc.	Found	Calc.	Found	Calc.	Found
**(1)**	**$C_{19}H_{20}OCl_2MnN_4S$**	**478.281**	Yellow	11.49	11.81	6.70	7.37	47.71	48.19	4.22	3.98	11.71	12.14
**(2)**	$C_{19}H_{20}OCl_2FeN_4S$	479.188	Red	11.65	12.56	6.69	6.61	47.62	47.87	4.21	4.00	11.69	12.11
**(3)**	$C_{19}H_{20}OCl_2NiN_4S$	482.036	Yellow	12.19	13.08	6.65	7.35	47.34	47.98	4.18	3.96	11.62	12.06
**(4)**	$C_{36}H_{32}Cl_2CuN_8S_2$	775.354	Black	8.19	8.52	8.27	8.34	55.77	55.89	3.64	3.66	14.45	14.61
**(5)**	$C_{72}H_{64}Cl_2ZnN_{16}S_4$	1418.064	Pink	4.61	4.85	9.05	8.48	60.99	60.51	3.98	3.96	15.80	15.49
